# Isothiocyanate Sulfur Atom as an Acceptor Site for Halogen-Bonded Cocrystallization of Werner Ni(II) Coordination Compounds and Perfluorinated Iodobenzenes

**DOI:** 10.1021/acs.cgd.4c00697

**Published:** 2024-08-26

**Authors:** Lidija Posavec, Dominik Cinčić

**Affiliations:** Department of Chemistry, Faculty of Science, University of Zagreb, Horvatovac 102a, 10000 Zagreb, Croatia

## Abstract

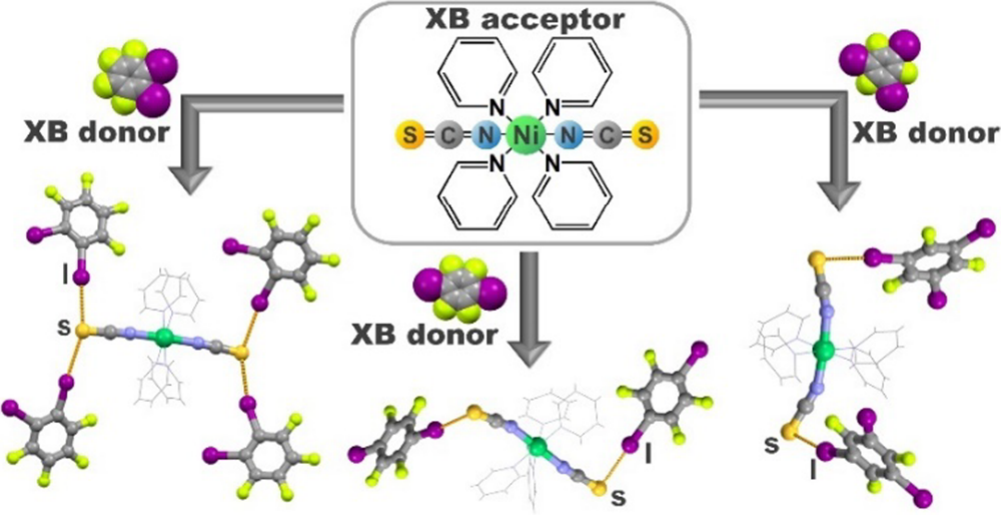

We explore the halogen bond acceptor potential of the isothiocyanate sulfur atom in the synthesis of cocrystals involving metal–organic building blocks by using Werner Ni(II) coordination compounds whose pendant isothiocyanate group enables halogen bonding. A series of 14 cocrystals involving octahedral Ni(L)_4_(NCS)_2_ coordination compounds (L = pyridine or 4-methylpyridine) has been prepared by both crystallization from solution and liquid-assisted grinding. The effectiveness of this strategy is demonstrated by the assembly of a large family of cocrystals involving five perfluorinated iodobenzenes. For both coordination compounds, we generally obtained one cocrystal with each donor; in one case, we obtained an additional two stoichiomorphs, and in another, we obtained three additional solvates. Single-crystal X-ray diffraction experiments revealed that building units in all cocrystals are connected via S···I halogen bonds involving the donor iodine atom and the isothiocyanate sulfur atom, which is an acceptor of two and, in some cases, even three halogen bonds. Consequently, both coordination compounds act as multitopic acceptors that can form multiple halogen bonds leading to the formation of one-, two-, and three-dimensional halogen-bonded architectures. The relative shortenings of S···I distances are from 7 to 15%, while the S···I–C angles are in the range from 160 to 180°.

## Abstract 2

Two metal−organic building blocks, octahedral Ni(L)_4_(NCS)_2_ coordination compounds (L = pyridine or 4-methylpyridine), were cocrystallized with a series of five perhalogenated benzenes as halogen bond donors. 14 cocrystals were obtained, allowing for a systematic study of the M-NCS···I halogen bond motif.

## Introduction

Extensive research in the field of halogen bonding (XB) throughout the last three decades has provided multiple insights into the nature of this intermolecular interaction.^1−3^ Due to properties such as directionality^4,5^ and easy tuning of the interaction strength,^6−9^ the halogen bond has provided multiple possibilities in the design of multicomponent systems.^10−14^ Most studies of halogen-bonded multicomponent materials have focused on cocrystals comprising organic molecules as halogen bond acceptors and perhalogenated compounds,^1,15−17^ while metal–organic building blocks have received much less attention.^18−20^ In comparison with pure organic building blocks, the reasons for using metal coordination compounds in the design of multicomponent systems are numerous; from interesting magnetic, electrical, and optical properties^21−23^ to the possibility of applying such systems in the process of catalysis.^24−26^ Coordination compounds can also provide a wide range of different geometries that are not available to simple organic molecules and can be easily modified by changing the metal center^27−30^ or by changing the ligands attached to the metal,^31,32^ thus enabling halogen bonds between the coordination compound and halogen bond donor molecules. In the literature, few approaches for the synthesis of halogen-bonded metal–organic cocrystals have been presented.^33^ The halogen bond acceptor functionality on the coordination compound can be introduced as an additional functional group on the periphery of the ligand (i.e., the pyridine nitrogen atom, the carbonyl or morpholinyl oxygen atom)^11,30−32,34,35^ or by using monovalent, inorganic anions, like halides or pseudohalides.^36−39^ Halide ligands, especially chloride ligands, show great acceptor ability in different coordination compounds, even when competing with other acceptor groups containing oxygen or nitrogen atoms.^40,41^ On the other hand, pseudohalide ligands have hardly been recognized as halogen bond acceptors, with most research done on cyanide coordination compounds.^43−45^ Furthermore, for both metal–organic and organic solids, the sulfur atom is significantly less studied as an acceptor species relative to the oxygen or nitrogen atom. According to available structural data in the Cambridge Structural Database (CSD),^46^ there are a total of 6880 data sets for the [S, I–X] motif (X being any atom and with an unspecified charge on S), and it was found that the S···I halogen bond is present in 811 data sets. A subset of these data corresponds to multicomponent crystals containing perhalogenated iodobenzenes (PHB) with 256 data sets, which is 17% of a total of 1627 data sets for structures with perhalogenated iodobenzenes. Of those, 127 data sets correspond to structures with a charged sulfur atom. Furthermore, for the [C=S, I–X] motif, there are 1053 data sets, of which 96 correspond to the [C=S, I_PHB_] motif. It was found that the C=S···I_PHB_ halogen bond is present in 94 data sets (98%, which represents the propensity of a particular acceptor species). When we narrowed the search to isothiocyanate sulfur as a halogen bond acceptor, it was found that the NCS···I halogen bond, including both neutral and charged sulfur atoms, is present in 167 data sets. Of those, only 28 correspond to structures with the M–NCS···I halogen bond motif (M being any metal atom) and only one structure is a multicomponent crystal that contains a perhalogenated halogen bond donor (1,4-diiodotetrafluorobenzene).^42^^,^^47^

In this work, we decided to explore the potential of the isothiocyanate ligand bonded to the metal atom as a reliable halogen bond acceptor species to form cocrystals containing metal–organic building blocks and perfluorinated iodobenzenes. As coordination compounds, we selected Werner coordination compounds that are mostly known for displaying inclusion phenomena.^48−50^ In general, these Werner coordination compounds are of the MX_2_L_4_ general formula, where M is a divalent metal cation, typically Ni(II), Co(II), Fe(II), Cu(II), or Mn(II), X is an anionic ligand (NCS^–^, CN^–^, NO_3_^–^, and NCO^–^), and L is a substituted pyridine or α-arylalkylamine.^51^ The remarkable chlathration ability of this type of complexes was first reported in 1957,^52^ and since then, many studies have been done on this type of coordination compounds.^53−56^ The most interesting feature of these compounds is the rotational freedom of the metal-N(pyridine) bond. For the substituted pyridine ligands, there is additional torsional flexibility at the substituent, which allows the coordination compound to adjust its shape to accommodate different guest molecules of varying shapes and sizes. This property can also be used for the separation of similar guest molecules (isomers), depending on the selectivity of Werner clathrates toward the components of a mixture and whether or not guest uptake/removal is reversible.^57−59^ For our research, we selected one of the most extensively studied Werner coordination compounds, Ni(4-methylpyridine)_4_(NCS)_2_ (**1**) and another compound very similar to it, Ni(pyridine)_4_(NCS)_2_ (**2**).^52,60^ Through modification of the pyridine ligand type within the metal coordination sphere, this type of coordination compound can exhibit different inclusion properties. For instance, coordination compound **2** exhibits only one type of clathrate due to the easy formation of a close-packed nonclathrate α-phase because it lacks substituents on the pyridine ring. Compound **1** is known to exist in two different polymorphic modifications: a microporous (β-form) phase that can change into a different type of clathrate when it interacts with guest molecules, and a densely packed, nonporous phase (α-form).^61^ Recently, this coordination compound has been studied in terms of shape-memory effects, since it possesses a porous polymorph. Through *p*-xylene vapor sorption studies, it was confirmed that compound **1** possesses properties of shape-memory material (SMM), meaning that it transforms to a new polymorphic morphology in response to an external stimulus and reverts to its original phase when subjected to a different external stimulus.^62^

**Scheme 1:**
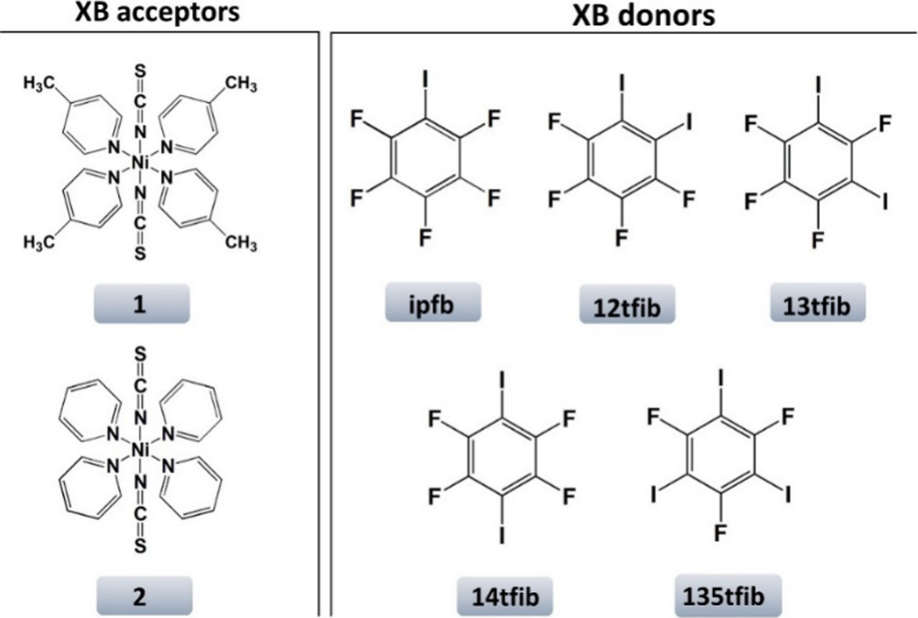
Molecular Schemes of Halogen Bond Acceptors and Halogen Bond Donors Used in This Study

As there has been no systematic research on this type of halogen bond acceptor, in this work, the selected coordination compounds **1** and **2** were cocrystallized with different halogen bond donors, perfluorinated iodobenzenes: 1,2-diiodotetrafluorobenzene (**12tfib**), 1,3-diiodotetrafluorobenzene (**13tfib**), 1,4-diiodotetrafluorobenzene (**14tfib**), 1,3,5-trifluoro-2,4,6-triiodobenzene (**135tfib**), and iodopentafluorobenzene (**ipfb**) (Scheme 1).

## Results and Discussion

Our screen for cocrystal synthesis was based on mechanochemical liquid-assisted grinding (LAG). As a means to explore the reactivity of solid reactants and the stoichiometric ratio, we first performed LAG of the reactants in stoichiometric ratios of 1:1 and 1:2 (coordination compound to halogen bond donor), respectively, and in the presence of a small amount of methanol or acetone. Grinding experiments were conducted in a Retsch MM200 mill using stainless steel jars under normal laboratory conditions (temperature ca. 25 °C, 40–60% relative humidity). Mechanochemical experiments were accompanied by crystallization from the solution in order to obtain bulk products and single crystals. Crystallization experiments were performed by dissolving a reactant mixture in an appropriate solvent with heating, followed by letting the solvent or solvent mixture cool down and evaporate at room temperature. The obtained products were characterized by thermogravimetric analysis (TGA), powder analysis (PXRD), and single-crystal X-ray diffraction (SCXRD) (see the Supporting Information). A total of 14 new cocrystals were synthesized and characterized. For both coordination compounds, we obtained one cocrystal with each donor; in the case of the (**1**)_2_(**14tfib**) cocrystal, we additionally prepared three solvates (with acetone, nitromethane, and acetonitrile), and for compounds **1** and **ipfb,** we isolated two stoichiomorphs. Molecular and crystal structure determination by SCXRD of the prepared solids revealed that in all cocrystals, metal–organic acceptors and halogen bond donors are connected via S···I halogen bonds involving a donor iodine atom and an isothiocyanate sulfur atom. Analysis of halogen bond parameters for each cocrystal confirmed the formation of halogen bonds, as the relative shortenings of S···I distances are from 7 to 14.7%, while the S···I–C angles are in the range from 160 to 180° (Table 1). It was shown that the isothiocyanate sulfur atom participates as an acceptor of at least two and, in some cases, three halogen bonds. Therefore, as both coordination compounds **1** and **2** contain two isothiocyanate groups, they act as multitopic acceptors that can form multiple halogen bonds in all prepared cocrystals (up to five in the cocrystal of compound **1** with **135tfib**).

**Table 1:** Halogen Bond Lengths (*d*), Angles (∠), and Relative Shortenings (R.S.) of X···A Distances of Compound **1** and **2** Cocrystals

cocrystal	D–X···A	*d*(X···A)/Å	∠ (D–X···A)/°	R.S.^a^/%
**(1)(12tfib)**	C4–I2···S1	3.329	169.5	11.9
	C3–I1···S2	3.357	172.9	11.1
**(1)(13tfib)_2_**	C18–I1···S1	3.306	171.3	12.6
**(1)(14tfib)_2_**	C19–I1···S1	3.336	176.9	11.8
	C16–I2···S1	3.516	172.0	7.0
**(1)_2_(14tfib)_3_(ACT)_2_**	C7–I1···S1	3.269	175.2	13.5
	C35–I2···S1	3.350	166.0	11.4
	C32–I3···S2	3.327	173.5	12.0
**(1)_2_(14tfib)_3_(NMT)_2_**	C6–I3···S1	3.237	173.6	14.4
	C3–I1···S2	3.397	172.0	10.1
	C9–I2···S2	3.305	178.4	12.6
**(1)_2_(14tfib)_3_(ACN)_2_**	C1–I1···S1	3.399	172.9	10.1
	C7–I3···S1	3.325	177.2	12.1
	C4–I2···S2	3.228	174.0	14.6
**(1)(135tfib)_2_**	C31–I3···S1	3.305	168.0	12.5
	C37–I6···S1	3.401	178.1	10.0
	C29–I2···S2	3.336	166.7	11.8
	C33–I4···S2	3.321	172.1	12.1
	C27–I1···S2	3.264	175.6	13.7
**(1)(ipfb)_2_**	C18–I1···S1	3.330	171.8	11.9
**(1)_2_(ipfb)_3_**	C12–I1···S1	3.339	174.6	11.6
	C4–I2···S2	3.380	176.8	10.6
	C14–I3···S2	3.326	173.9	12.0
**(2)(12tfib)_2_**	C6–I2···S1	3.387	165.2	10.3
	C36–I8···S1	3.313	163.4	12.35
	C13–I3···S2	3.345	173.5	11.5
	C18–I5···S2	3.378	168.2	10.6
	C5–I2···S3	3.332	171.7	11.8
	C14–I4···S3	3.393	165.8	10.3
	C24–I7···S4	3.291	176.8	12.9
	C19–I6···S4	3.394	174.0	10.2
**(2)_2_(13tfib)_3_**	C17–I6···S1	3.408	169.0	9.8
	C6–I1···S1	3.319	173.2	12.2
	C13–I5···S2	3.322	166.3	12.1
	C2–I2···S3	3.292	173.7	12.9
	C12–I3···S4	3.384	168.4	10.5
	C10–I4···S4	3.486	163.0	7.7
**(2)_2_(14tfib)_3_**	C3–I3···S1	3.366	173.9	10.9
	C16–I2···S2	3.277	173.6	13.3
**(2)(135tfib)**	C27–I2···S1	3.432	165.0	9.2
	C25–I1···S2	3.263	175.2	13.7
**(2)(ipfb)_2_**	C19–I1···S1	3.225	177.5	14.7

a R.S. = 1 – *d*(X···A)/[*r*_vdW_(X) + *r*_vdW_(A)].

**Figure 1:**
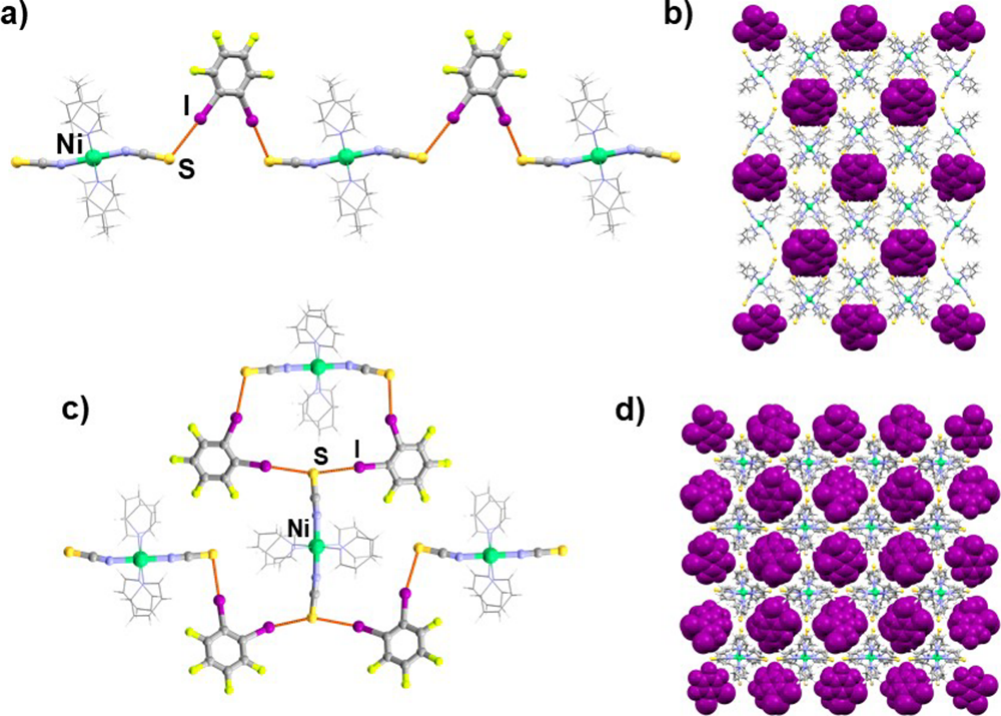
(a) Halogen-bonded chain in the (**1**)(**12tfib**) cocrystal, (b) crystal packing of the (**1**)(**12tfib**), (c) halogen-bonded layers in (**2**)(**12tfib**)_2_ cocrystal, and (d) crystal packing of the (**2**)(**12tfib**)_2_ (donor molecules are shown in purple and with a spacefill model).

The possibility of forming multiple halogen bonds is a result of the great acceptor ability of the thiocyanate sulfur atom. First, in contrast to coordination compounds containing simple ligands that were previously reported as halogen bond acceptor species (for example, −CN, −Cl), the sulfur atom is more distant from the metal center and other ligands, making it sterically more accessible for halogen bonding. The second advantage of the isothiocyanate group is its flexibility and the ability to bend in order to participate in halogen bonding. Isothiocyanate groups present in metal–organic units in the prepared cocrystals are bent at distinctly different angles. The isothiocyanate group’s bending (Ni–N–C) angles in cocrystals of compound **1** are 137–177°, and in cocrystals of compound **2**, they are 150–174° (see Tables S2 and S3). Furthermore, the geometry of the selected coordination compounds is also a relevant factor when it comes to the formation of multiple halogen bonds. The most prominent characteristic of the studied Werner coordination compounds is the rotational freedom of the metal–N(pyridine) bond. This facilitates easy access of the isothiocyanate group to the donor molecules and allows the coordination compound to reshape itself for optimal crystal packing. In all cocrystals, the molecular structure of coordination compounds is in good agreement with those of pure **1** and **2,** reported as *trans* isomers. The Ni(II) atom is coordinated by four nitrogen atoms from pyridine molecules and two isothiocyanate groups, forming a structure with a distorted octahedral geometry. The only significant difference in coordination compounds is in the conformation of the pyridine ligands present on the metal center. In pure compound **1** pyridine ligands are arranged in the so-called propeller conformation (where opposite pyridine rings are orthogonal), while in pure compound **2,** the opposite pyridine rings are planar. However, except for the (**1**)(**14tfib**)_2_ cocrystal, in all other cocrystals of compounds **1** and **2**, the pyridine rings adopt a propeller conformation. The rotational freedom of the Ni–N_pyr_ bond allows the pyridine rings in various cocrystals to rotate at different angles. To quantify the flexibility of metal–organic units in the synthesized cocrystals, we analyzed angles between the plane of pyridine rings and the plane parallel to the metal center and the four nitrogen atoms from the pyridine ligands (see Tables S4 and S5). For compound **1**, these angles range from 36 to 77°, and for compound **2**, from 43 to 65°. Although compounds **1** and **2** differ only in one methyl group on the pyridine ligand, they nevertheless form very different cocrystals. The topicity and geometry of the donor molecule, as well as the geometric properties of the coordination compound, influence the stoichiometry and crystal packing. Pyridine ligands, which are present in compound **2**, have a propensity to cluster closely in the crystal structure. On the other hand, compound **1** has additional torsional flexibility at the methyl substituent and can easily adjust its shape to accommodate different donor molecules. Compound **1** in cocrystals tends to form S···I halogen bonds with more donor molecules compared to compound **2** (Table 1). Exceptions are cocrystals with ditopic donor molecules that have donor atoms at a bent angle (**13tfib** and **12tfib**), which form multiple halogen bonds with coordination compound **2** (Table 2). The reason behind this could be the absence of a methyl group on pyridine rings, which geometrically enables bridging two metal coordination compounds with at least one donor molecule (Figure 1c).

**Table 2:** Decomposition Temperatures (*T*_d_) of Compound **1** and **2** Cocrystals Determined by TGA Experiments

cocrystal	*T*_d_/°C
**(1)(12tfib)**	97
**(2)(12tfib)_2_**	90
**(1)(13tfib)_2_**	70
**(2)_2_(13tfib)_3_**	80
**(1)_2_(14tfib)_3_(ACT)_2_**	67
**(1)_2_(14tfib)_3_(NMT)_2_**	65
**(1)_2_(14tfib)_3_(ACN)_2_**	62
**(1)(14tfib)_2_**	105
**(2)_2_(14tfib)_3_**	96
**(1)(135tfib)_2_**	115
**(2)(135tfib)**	111
**(1)(ipfb)_2_**	69
**(1)_2_(ipfb)_3_**	68
**(2)(ipfb)_2_**	48

Thermal analysis revealed that the prepared halogen-bonded cocrystals decompose in two steps upon heating. By evaluating the inflection point temperature for the step of thermal degradation (*T*_d_, Table 2; also see the Supporting Information), it was found that thermal stability is correlated with the nature of the halogen bond donor molecule. The cocrystals with halogen bond donors **12tfib**, **14tfib,** and **135tfib** exhibit thermal degradation in the range of ca. 90–115 °C, and those with **ipfb** and **13tfib** decompose between 50 and 80 °C (see the Supporting Information). The thermal degradation temperatures are remarkably similar in spite of the different supramolecular architectures and significant differences in halogen bond donor melting points. **Ipfb** and **13tfib** donor molecules are liquids at room temperature with melting points at ∼ −31 and ∼23 °C, while **12tfib**, **14tfib,** and **135tfib** are solids with melting points at ∼50, ∼108, and ∼155 °C, respectively. Pure compound **1** decomposes at ∼90 °C, while compound **2** decomposes at ∼70 °C. Consequently, in most cases, cocrystals of compound **1** show higher degradation temperatures than cocrystals of **2**. Cocrystal solvates are an exception to this principle, as they exhibit expectedly much lower temperatures of thermal degradation than nonsolvate forms.

**Figure 2:**
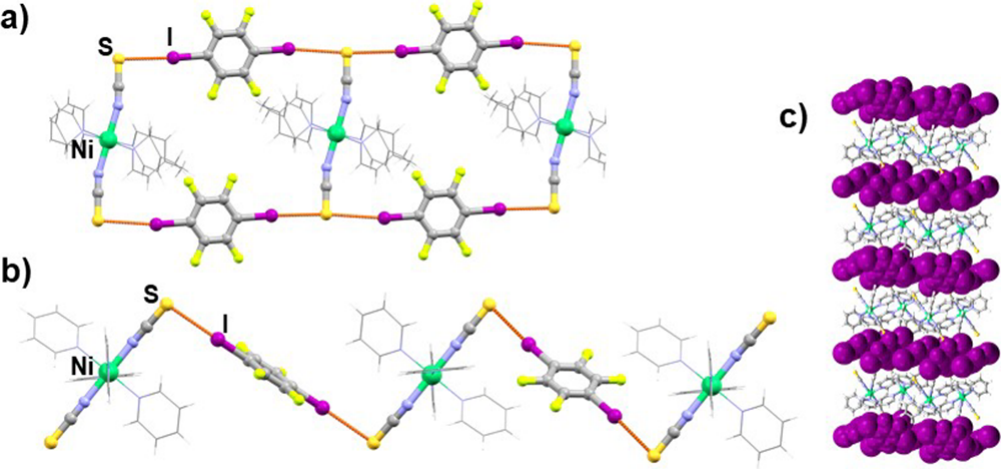
(a) Halogen-bonded double chain in the (**1**)(**14tfib**)_2_ cocrystal, (b) halogen-bonded chain in the (**2**)_2_(**14tfib**)_3_ cocrystal, and (c) crystal packing of the (**2**)_2_(**14tfib**)_3_ (donor molecules are shown in purple and with a spacefill model).

The topologies of the halogen bond networks in most cocrystals are associated with the topicity and geometry of the halogen bond donor. As expected, the monotopic donor (**ipfb**) forms discrete 1:2 halogen-bonded complexes with both coordination compounds. We also isolated one stoichiomorph with 2:3 stoichiometry that also exhibits discrete halogen-bonded units. Cocrystals based on **12tfib**, **135tfib**, and **14tfib** exhibit higher dimensionality, halogen-bonded chains, and layers. In (**1**)(**12tfib**), (**1**)(**14tfib**)_2_, (**2**)_2_(**14tfib**)_3_, and (**2**)(**135tfib**) cocrystals, the coordination compound and donor molecules form halogen-bonded chains, whereas in (**1**)(**135tfib**)_2_, (**2**)(**12tfib**)_2_, and the obtained cocrystal solvates, the coordination compound and donor molecules form complex halogen-bonded layers. The ditopic donor molecule **13tfib** forms cocrystals with both coordination compounds but different halogen bonding topologies. In the cocrystal with compound **1**, discrete halogen-bonded units are formed, while with compound **2,** donor molecules form complex halogen-bonded layers. The crystal packing of all obtained cocrystals is similar, with metal–organic units closely packed together and the donor molecules stacked in another layer. The crystal structures of cocrystals can be described in terms of alternating layers/chains of metal–organic units and donor molecules (Figures 1b,d, 2c, 4b,d, 5b,d, 6b,e).

Cocrystallization of ditopic donor **12tfib** with compounds **1** and **2** resulted in the formation of cocrystals with different stoichiometries and supramolecular architectures. In the structure of (**1**)(**12tfib**), each metal–organic molecule is connected by I···S halogen bonds between isothiocyanate sulfur atoms and two **12tfib** molecules, with **12tfib** acting as a ditopic halogen bond donor. This results in halogen-bonded chains (Figure 1a), which are further connected by C–H···S hydrogen bonds into a 3D network, *d*(C30···S2) = 3.826 Å. In the structure of (**2**)(**12tfib**)_2_, the asymmetric unit contains four crystallographically independent **12tfib** molecules and two metal–organic molecules. Each metal–organic unit participates in halogen bonding, forming four I···S halogen bonds between isothiocyanate sulfur atoms and **12tfib** molecules. This results in a 2D halogen-bonded network (Figure 1c). The layers are further connected to a 3D network by C–H···F contacts. In comparison with (**1**)(**12tfib**), it can be assumed that multiple I···S halogen bonds of metal–organic units are present due to the different coordination compound periphery, i.e., due to the less sterically complicated geometry of the compound **2**. This allows **12tfib** molecules with a bent geometry (60° angle of propagation) to have easier access to the isothiocyanate group and the acceptor sulfur atom.

Cocrystallization of the most extensively used ditopic perfluorinated halogen bond donor, **14tfib**, with compounds **1** and **2** yielded two cocrystals. Similar to (**2**)(**12tfib**)_2_, in (**1**)(**14tfib**)_2_, each metal–organic molecule participates in halogen bonding, forming four I···S halogen bonds between isothiocyanate sulfur atoms and **14tfib** molecules. This results in ladder-like halogen-bonded chains (Figure 2a), which are further connected into a 3D network by C–H···F contacts. The cocrystals of compound **2** and **14tfib** molecules exhibit 2:3 acceptor to donor stoichiometry. The asymmetric unit contains three crystallographically independent **14tfib** molecules, of which two exhibit similar supramolecular bonding. They participate as ditopic donors in I···S halogen bonds, with metal–organic molecules acting as ditopic halogen bond acceptors and forming halogen-bonded chains (Figure 2b). Surprisingly, the third crystallographically independent **14tfib** molecule is not halogen-bonded at all, neither with neighboring metal–organic units nor with **14tfib** molecules. This donor molecule plays the role of a void filler within the crystal packing. The chains of halogen-bonded molecules are connected into layers by C–H···S contacts (*d*(C14···S1) = 3.784 Å), which are further linked into a 3D network by C–H···F contacts.

**Figure 3:**
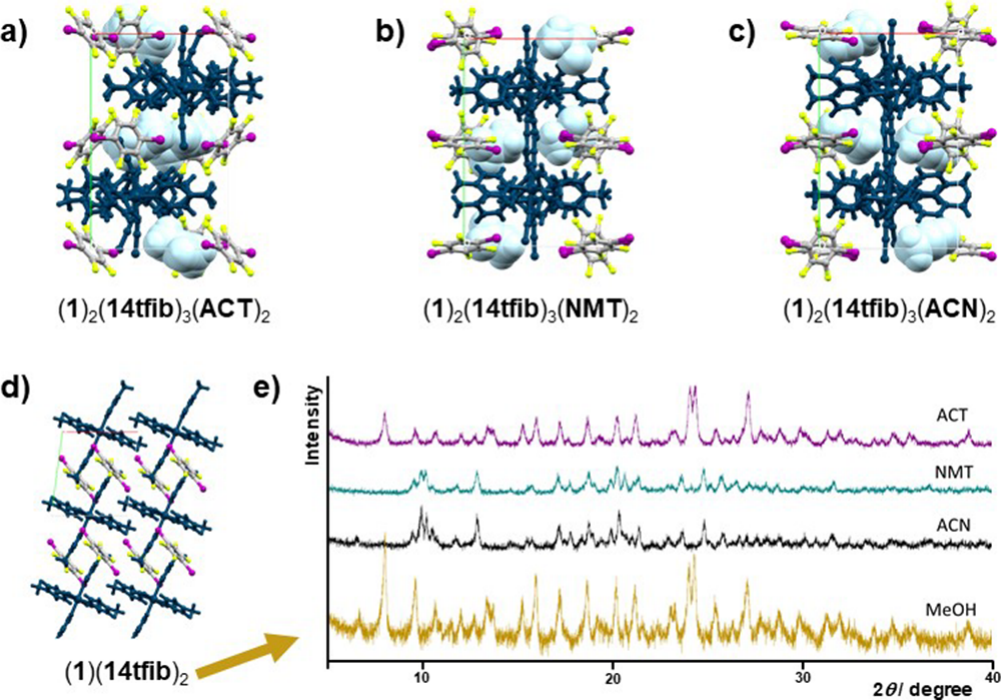
Crystal packing of cocrystal solvates and cocrystal obtained by the crystallization of **1** and **14tfib** from (a) acetone, (b) nitromethane, (c) acetonitrile, (d) methanol, and (e) powder patterns obtained by the milling of the same coformers with previously mentioned liquid additives. The coordination compound is colored dark blue, while the solvent molecules are shown in light blue color.

Furthermore, cocrystallization experiments from the solution involving compounds **1** and **14tfib** additionally resulted in the formation of three solvates of (**1**)(**14tfib**)_2_, with nitromethane, acetone, or acetonitrile. Two solvates out of these three (with nitromethane and acetonitrile) were also prepared by liquid-assisted grinding. On the other hand, mechanochemical experiments using a small amount of acetone yielded the nonsolvate cocrystal (**1**)(**14tfib**)_2_ (Figure 3). The reason for that could be explained by solvate instability under milling conditions and the high vapor pressure of acetone. Interestingly, the same cocrystallization experiments using the above solvents with compound **2** did not result in the formation of solvates.

In terms of halogen bonding, the supramolecular architectures of all three solvates are comparable and very similar. In all solvates, each metal–organic molecule participates in halogen bonding forming three I···S halogen bonds between isothiocyanate sulfur atoms and **14tfib** molecules. One isothiocyanate sulfur atom participates in two I···S halogen bonds, while the other is an acceptor of only one I···S halogen bond. This type of halogen bonding connects the metal–organic molecules and **14tfib** molecules into 2D layers which are further connected by C–H···F contacts into a 3D network. Solvent molecules present in the crystal structures are connected to metal–organic units by C–H···O/N hydrogen bonds (C–H···O hydrogen bond in the acetone solvate, *d*(C10···O1) = 3.396 Å; C–H···N hydrogen bond in the acetonitrile solvate, *d*(C27···N7) = 3.674 Å and C–H···O hydrogen bond in the nitromethane solvate *d*(C27···O1) = 3.312 Å).

**Figure 4:**
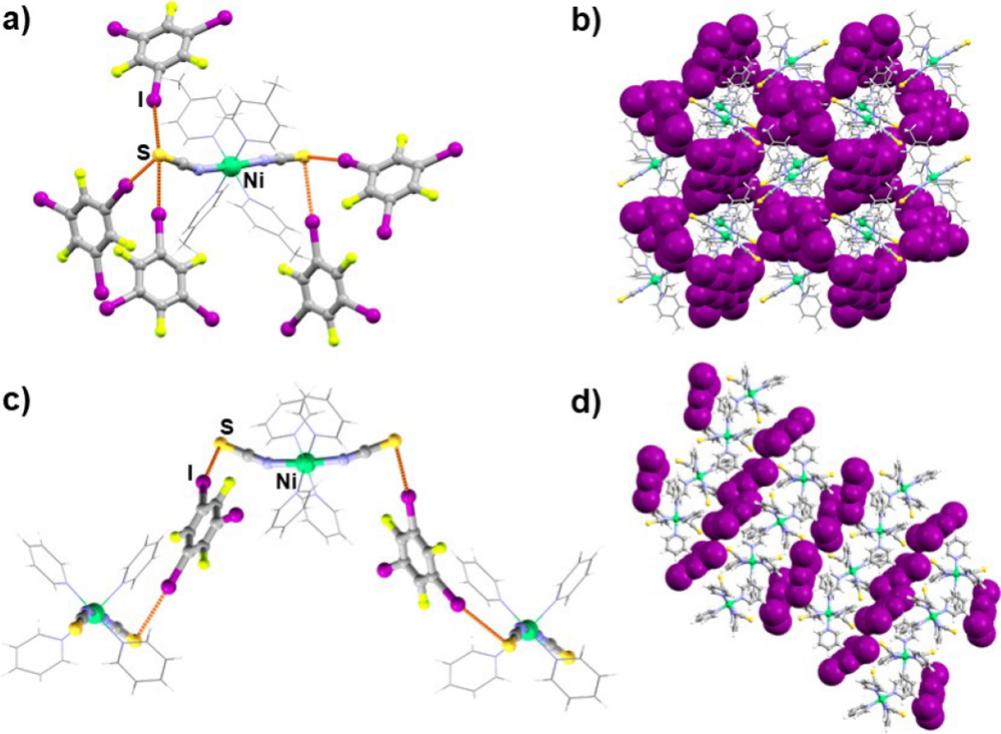
(a) Discrete halogen-bonded unit in (**1**)(**135tfib**)_2_ cocrystal, (b) crystal packing of the (**1**)(**135tfib**)_2_, (c) halogen-bonded chain present in (**2**)(**135tfib**) cocrystal, and (d) crystal packing of the (**2**)(**135tfib**) (donor molecules are shown in purple and with a spacefill model).

As with previously described cocrystals, cocrystallization of **135tfib** with compounds **1** and **2** resulted in cocrystals with different stoichiometries and significantly different supramolecular architectures. However, in the (**1**)(**135tfib**)_2_ cocrystal, each metal–organic molecule acts as a pentatopic halogen bond acceptor and, therefore, participates in halogen bonding forming five I···S halogen bonds between isothiocyanate sulfur atoms and **135tfib** molecules. One isothiocyanate sulfur atom participates in three I···S halogen bonds and the other one in two (Figure 4a). This results in complex halogen-bonded layers, which are further connected by C–H···I contacts (*d*(C14···I2) = 3.911 Å) into a 3D structure. The cocrystal (**2**)(**135tfib**), exhibits a very similar halogen-bonded architecture as the (**1**)(**12tfib**) and (**2**)(**14tfib**) cocrystals. Each metal–organic molecule is connected with two **135tfib** molecules by I···S halogen bonds, with **135tfib** acting as a ditopic halogen bond donor, forming halogen-bonded chains (Figure 4c), which are further connected by C–H···S hydrogen bonds (*d*(C16···S2) = 3.687 Å) and I···I contacts, (*d*(I3···I1) = 4.051 Å) into a 3D network.

**Figure 5:**
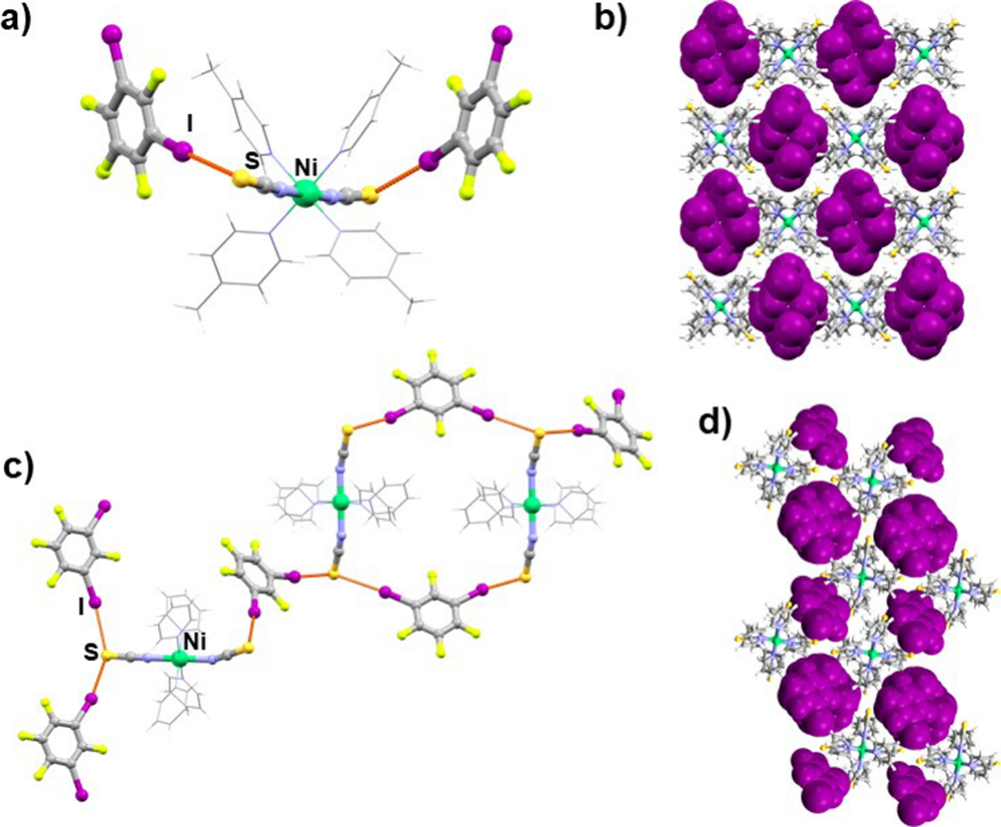
(a) Discrete halogen-bonded unit in (**1**)(**13tfib**)_2_ cocrystal, (b) crystal packing of the (**1**)(**13tfib**)_2_, (c) halogen-bonded layer present in (**2**)_2_(**13tfib**)_3_ cocrystal, and (d) crystal packing of the (**2**)_2_(**13tfib**)_3_ (donor molecules are shown in purple and with a spacefill model).

The prepared **13tfib** cocrystals with compounds **1** and **2** are interesting, since we obtained cocrystals of different stoichiometries and topicities of the halogen bond donor molecule. Cocrystallization of compound **1** with **13tfib** resulted in the formation of discrete halogen-bonded units (Figure 5a), since both **13tfib** molecules act as monotopic halogen bond donors. Each metal–organic molecule participates in halogen bonding, forming two I···S halogen bonds with **13tfib** molecules. Discrete halogen-bonded units are further connected into layers via C–I···F contacts, which are further connected into a 3D network by C–H···S contacts; *d*(C5···S2) = 3.578 Å. In the structure of (**2**)_2_(**13tfib**)_3_, the connectivity between **13tfib** molecules and metal–organic molecules is significantly different. The asymmetric unit contains three crystallographically independent **13tfib** molecules and two metal–organic units. Each metal–organic unit participates in three I···S halogen bonds between isothiocyanate sulfur atoms and **13tfib** molecules. One isothiocyanate sulfur atom participates in two I···S halogen bonds, while the other one participates in one halogen bond. This results in a 2D halogen-bonded network. The layers are further connected into a 3D network by C–H···F and C–H···S contacts (Figure 5c).

**Figure 6:**
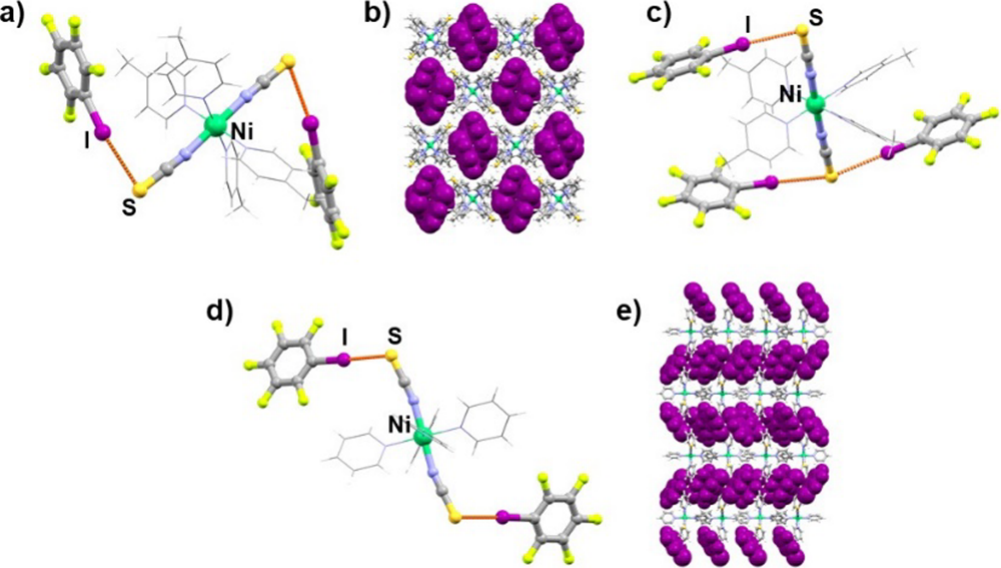
(a) Discrete halogen-bonded unit in the (**1**)(**ipfb**)_2_ cocrystal, (b) crystal packing of the (**1**)(**ipfb**)_2_, (c) discrete halogen-bonded unit in the (**1**)_2_(**ipfb**)_3_ cocrystal, (d) discrete halogen-bonded unit in the (**2**)(**ipfb**)_2_ cocrystal, (d) crystal packing of the (**2**)(**ipfb**)_2_ (donor molecules are shown in purple and with a spacefill model).

Cocrystallization of compound **1** with **ipfb** yielded two cocrystals of different stoichiometries, in 1:2 and 2:3 stoichiometric ratios. The (**1**)(**ipfb**)_2_ cocrystal exhibits structural motifs very similar to those of the (**1**)(**13tfib**)_2_ cocrystal where each metal–organic molecule participates in halogen bonding with two **ipfb** molecules by I···S halogen bonds, forming discrete halogen-bonded units (Figure 6a). Halogen-bonded trimers are further connected in two and three dimensions by C–H···S contacts, *d*(C3···S1) = 3.660 Å and *d*(C11···S1) = 3.820 Å. When the crystal packings of these two cocrystals are compared, they are almost of identical supramolecular architecture, the only difference being the presence of a different donor molecule. Another stoichiomorph, the (**1**)_2_(**ipfb**)_3_ cocrystal, was obtained by both changing the crystallization conditions and changing the stoichiometric ratio in a mechanochemical reaction. The asymmetric unit contains three crystallographically independent **ipfb** molecules and two metal–organic molecules of which only one participates in halogen bonding with three **ipfb** molecules by I···S halogen bonds. The second crystallographically independent metal–organic unit participates only in C–H···S contacts with other neighboring metal–organic units (Figure 6c). Like in (**1**)(**ipfb**)_2_, in the (**2**)(**ipfb**)_2_ structure, each metal–organic molecule participates in halogen bonding with two halogen bond donor molecules by I···S halogen bonds, forming discrete halogen-bonded units (Figure 6d). Halogen-bonded trimers are further connected into a chain by C–H···F contacts, *d*(C8···F2) = 3.251 Å, which are further connected in the second and third dimension by C–H···S contacts, *d*(C13···S1) = 3.719 Å.

**Figure 7:**
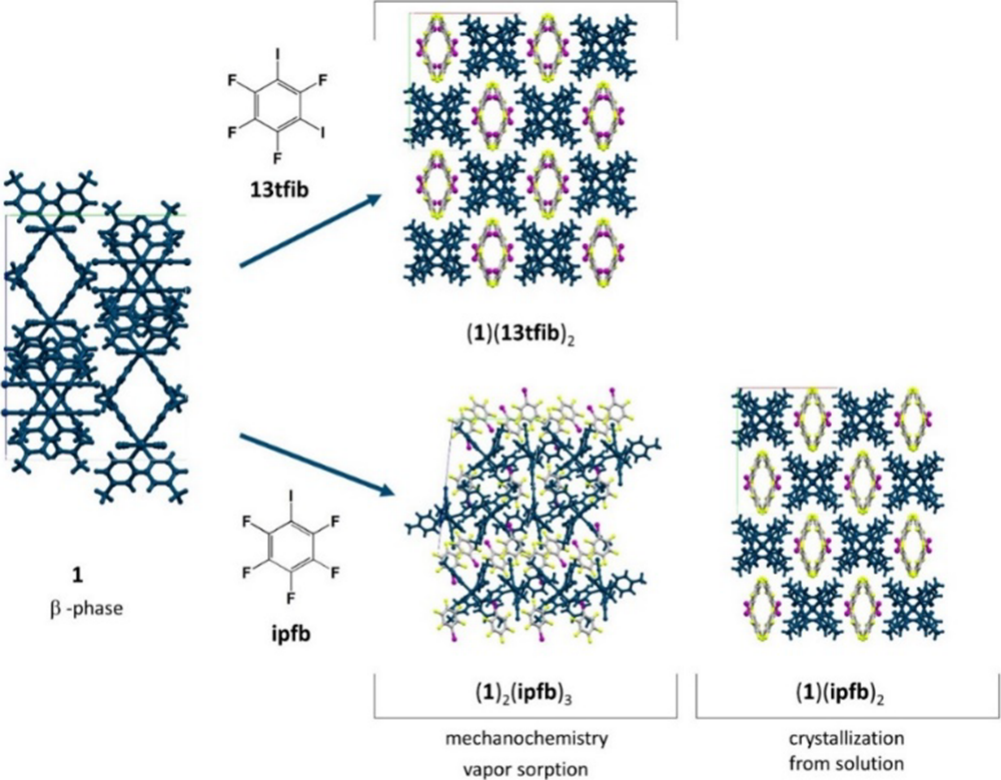
Crystal packing of cocrystals obtained from **1** and liquid donor molecules (**13tfib** and **ipfb**) by using vapor sorption experiments or mechanochemical synthesis.

Finally, due to similar supramolecular architectures of cocrystals with volatile donors, **ipfb** and **13tfib**, we were able to compare the reactivity of compounds **1** and **2** with donors using vapor sorption experiments. Aging of compound **1** in an atmosphere of **13tfib** for 6 h at 70 °C and then for 2 days at room temperature afforded the (**1**)(**13tfib**)_2_ cocrystal, identical to the cocrystal prepared by the solution method and mechanochemical experiments. Likewise, an aging experiment of compound **1** in an atmosphere of **ipfb** afforded a cocrystal product identical to the one prepared by the mechanochemical experiment, (**1**)_2_(**ipfb**)_3_, which is probably thermodynamically more stable than the (**1**)(**ipfb**)_2_ cocrystal obtained by the solution method (Figures S28 and S29). Interestingly, aging experiments with compound **2** in the atmosphere of the same donor molecules at the same conditions did not result in the formation of cocrystals. A probable explanation for this outcome is the microporosity of the starting compound **1** (β-phase), which enables the incorporation of donor molecules in the structure and the formation of cocrystals (Figure 7).

## Conclusions

The family of 14 halogen-bonded cocrystals prepared herein demonstrates that nickel(II) coordination compounds with isothiocyanate ions can be used as reliable halogen bond acceptors. In all cocrystals, the main acceptor atom is the isothiocyanate sulfur atom, which shows great potential for halogen bonding, forming up to three halogen bonds with perhalogenated benzenes. Therefore, as both coordination compounds contain two isothiocyanate groups, they act as multitopic acceptors that can form multiple halogen bonds. In this particular instance, coordination compound **1** participates in more halogen bonds than compound **2** in the majority of cocrystals. Since coordination compound **2** molecules, which contain pyridine ligands, tend to cluster closely in the crystal structure, fewer donor molecules can access the sulfur atom. Only in cocrystals with donor molecules that have donor atoms bent (**13tfib** and **12tfib**) is the situation reversed, with more halogen bonds being formed with coordination compound **2**. This may be due to the fact that pyridine rings lack a methyl group, which allows donor molecules to geometrically bridge two metal coordination compounds. By exploiting the geometric properties of the herein-used Werner coordination compounds, different multicomponent systems can also be synthesized (stoichiomorphs and cocrystal solvates). The rotational freedom of pyridine substituents and the flexibility of the isothiocyanate group enable these coordination compounds to adjust their shape to accommodate additional halogen bond donor molecules. By combining the structural properties of coordination compounds with halogen bond acceptor properties, additional ways of synthesis can also be used (vapor sorption experiments for microporous coordination compounds). This family of cocrystals demonstrates the uncovered potential of metal–organic systems, which can be used as building blocks in crystal engineering, providing multiple opportunities for synthesis.

## Experimental Section

### Synthesis

All substances, except coordination compounds **1** and **2**, were purchased from commercial sources and used without further purification. Coordination compounds **1** and **2** were synthesized according to the procedure described by Schaeffer et al.^52^

### Mechanochemical Synthesis

Cocrystal synthesis was performed by grinding mixtures of coordination compound **1** or **2** with a selected halogen bond donor. A mixture of reactants (up to 100 mg) was placed in a 10 mL stainless steel jar along with 20 μL of methanol or acetone and two stainless steel balls measuring 7 mm in diameter. The reaction mixture was then milled for 60 min in a Retsch MM200 Shaker Mill operating at 25 Hz, under normal laboratory conditions (temperature approximately 25 °C, 40–60% relative humidity). The resulting powders were characterized by powder X-ray diffraction. Details on mechanochemical experiments are given in the Supporting Information.

### Vapor Sorption Experiments

For the synthesis of cocrystals containing liquid donor molecules **13tfib** and **ipfb** and coordination compound **1**, vapor sorption experiments were performed. The experiments were conducted in closed glass vials with caps. The powder of coordination compound **1** was placed in an open Eppendorf tube, and the liquid (100 μL of selected perhalogenated benzene) was placed at the bottom of the glass vial. The glass vials were left at 70 °C for 6 h and then left at room temperature for 2 days. The resulting powders were characterized by powder X-ray diffraction. The PXRD patterns are given in Figures S28 and S29.

### Single Crystal Preparation

Crystals suitable for single-crystal X-ray diffraction experiments were prepared by crystallization, mostly from methanol or a mixture of methanol with other solvents. Approximately 20 mg of a mixture of coordination compound **1** or **2** with a selected halogen bond donor was dissolved in 5.00 mL of the chosen solvent or mixture of solvents with heating. The crystals were obtained by the slow evaporation of the solvent at room temperature after a few days. Details of crystallization experiments are given in the Supporting Information.

### Powder X-ray Diffraction

PXRD experiments were performed on a Malvern PANalytical Aeris Research Edition X-ray diffractometer with Cu*K*_α1_ (1.54056 Å) radiation at 15 mA and 40 kV. The scattered intensities were measured with a line detector. The angular range was from 5 to 40° (2θ)) with integrated steps of 0.0054332° (2θ)), and the measuring time was 10.2 s per step. Data analysis was performed using the program package Data Viewer.^63^ PXRD patterns are given in Figures S18–S29.

### Single-Crystal X-ray Diffraction

The crystal and molecular structures of the prepared cocrystals, cocrystal solvates, and stoichiomorphs were determined by single-crystal X-ray diffraction. Diffraction measurements were made on a Rigaku Synergy XtaLAB X-ray diffractometer with graphite-monochromated Mo*K*_α_ (λ = 0.71073 Å) radiation. The data sets were collected using the ω scan mode over a 2θ range up to 64° (Synergy XtaLAB). Programs CrysAlis CCD, CrysAlis RED, and CrysAlisPro were employed for data collection, cell refinement, and data reduction, respectively.^64^ The structures were solved by direct methods and refined using the SHELXT, SHELXS, and SHELXL programs, respectively.^65,66^ Structural refinement was performed on *F*^2^ by using all data. Non-hydrogen atoms were refined anisotropically and hydrogen atoms were placed in calculated positions and treated as riding on their parent atoms. All calculations were performed using the WINGX crystallographic suite of programs.^67^ Molecular structures of compounds and their molecular packing projections were prepared using Mercury 2022.3.0.^68^ Details of data collection and crystal structure refinement are listed in Table S1. Molecular structures showing the atom-labeling shemes are given in Figures S1–14.

### Thermogravimetric Analysis

TGA measurements were performed on a Mettler-Toledo TGA/DSC 3+ module. The samples were placed in open 70 μL alumina pans and heated from 30 to 800 °C for coordination compounds **1**, **2,** and prepared cocrystals at a rate of 10 °C min^–1^ under nitrogen flow of 50 mL min^–1^. The data collection and analysis were performed using the program package STARe Software 15.00.^69^ TGA curves are given in Figures S30–S45.

## Supporting Information Available

Experimental details, including syntheses, single crystal diffraction data, PXRD patterns, and TGA curves (PDF)
