# Supplementary material for: Isothiocyanate Sulfur Atom as an Acceptor Site for Halogen-Bonded Cocrystallization of Werner Ni(II) Coordination Compounds and Perfluorinated Iodobenzenes
Source: Cryst Growth Des. 2024 Aug 26;24(18):7514–23. doi: 10.1021/acs.cgd.4c00697 (PMC11421206; doi:10.1021/acs.cgd.4c00697)
Supplement: Supplementary file 1 — cg4c00697_si_001.pdf [file cg4c00697_si_001.pdf]

SUPPORTING INFORMATION

The Isothiocyanate Sulfur Atom as an Acceptor Site for Halogen-Bonded Cocrystallization of Werner Ni(II) Coordination Compounds and Perfluorinated Iodobenzenes

Lidija Posavec, Dominik Cinčić*

Department of Chemistry, Faculty of Science, University of Zagreb, Horvatovac 102a, HR-10000 Zagreb, Croatia

E-mail: dominik@chem.pmf.hr
Fax: +385 1 4606 341
Tel: +385 1 4606 362

Table of Contents

Experimental details	Mechanochemical syntheses, solution syntheses, thermal analysis, powder X-ray diffraction experiments, single-crystal X-ray diffraction experiments	*3*
Table S1.	Crystal data and refinement details for the prepared cocrystals.	9
Table S2.	Rotation angles (∠) of isothiocyanate group (Ni-NCS) present in the herein prepared cocrystals and starting coordination compound **1**	16
Table S3.	Rotation angles (∠) of isothiocyanate group (Ni-NCS) present in the herein prepared cocrystals and starting coordination compound **2**	16
Table S4.	Rotation angles (∠) of pyridine rings present in the herein prepared cocrystals and starting coordination compound **1**	17
Table S5.	Rotation angles (∠) of pyridine rings present in the herein prepared cocrystals and starting coordination compound **2**	18
Figure S1.	Molecular structure of (**1**)(**12tfib**) showing the atom-labeling scheme. Displacement ellipsoids are drawn at the 50 % probability level, and H atoms are shown as small spheres of arbitrary radius.	19
Figure S2.	Molecular structure of (**1**)(**13tfib**)$_2$ showing the atom-labeling scheme. Displacement ellipsoids are drawn at the 50 % probability level, and H atoms are shown as small spheres of arbitrary radius.	19
Figure S3.	Molecular structure of (**1**)(**14tfib**)$_2$ showing the atom-labeling scheme. Displacement ellipsoids are drawn at the 50 % probability level, and H atoms are shown as small spheres of arbitrary radius.	20
Figure S4.	Molecular structure of (**1**)(**135tfib**)$_2$ showing the atom-labeling scheme. Displacement ellipsoids are drawn at the 50 % probability level, and H atoms are shown as small spheres of arbitrary radius.	20
Figure S5.	Molecular structure of (**1**)(**ipfb**)$_2$ showing the atom-labeling scheme. Displacement ellipsoids are drawn at the 50 % probability level, and H atoms are shown as small spheres of arbitrary radius.	21
Figure S6.	Molecular structure of (**2**)(**12tfib**) showing the atom-labeling scheme. Displacement ellipsoids are drawn at the 50 % probability level, and H atoms are shown as small spheres of arbitrary radius.	21
Figure S7.	Molecular structure of (**2**)$_2$(**13tfib**)$_3$ showing the atom-labeling scheme. Displacement ellipsoids are drawn at the 50 % probability level, and H atoms are shown as small spheres of arbitrary radius.	22
Figure S8.	Molecular structure of (**2**)$_2$(**14tfib**)$_3$ showing the atom-labeling scheme. Displacement ellipsoids are drawn at the 50 % probability level, and H atoms are shown as small spheres of arbitrary radius.	22

Figure S9.	Molecular structure of (**2**)(**135tfib**)$_2$ showing the atom-labeling scheme. Displacement ellipsoids are drawn at the 50 % probability level, and H atoms are shown as small spheres of arbitrary radius.	23
Figure S10.	Molecular structure of (**2**)(**ipfb**)$_2$ showing the atom-labeling scheme. Displacement ellipsoids are drawn at the 50 % probability level, and H atoms are shown as small spheres of arbitrary radius.	23
Figure S11.	Molecular structure of (**1**)$_2$(**14tfib**)$_3$(**ACT**)$_2$ showing the atom-labeling scheme. Displacement ellipsoids are drawn at the 50 % probability level, and H atoms are shown as small spheres of arbitrary radius.	24
Figure S12.	Molecular structure of (**1**)$_2$(**14tfib**)$_3$(**ACN**)$_2$ showing the atom-labeling scheme. Displacement ellipsoids are drawn at the 50 % probability level, and H atoms are shown as small spheres of arbitrary radius.	24
Figure S13.	Molecular structure of (**1**)$_2$(**14tfib**)$_3$(**NMT**)$_2$ showing the atom-labeling scheme. Displacement ellipsoids are drawn at the 50 % probability level, and H atoms are shown as small spheres of arbitrary radius.	25
Figure S14.	Molecular structure of (**1**)$_2$(**ipfb**)$_3$ showing the atom-labeling scheme. Displacement ellipsoids are drawn at the 50 % probability level, and H atoms are shown as small spheres of arbitrary radius.	25
Figure S15.	PXRD pattern of **12tfib**.	26
Figure S16.	PXRD pattern of **14tfib**.	26
Figure S17.	PXRD pattern of **135tfib**.	27
Figure S18.	PXRD patterns of: a) **12tfib**, b) **1**, c) product obtained by grinding **12tfib** and **1** in a 1:1 stoichiometric ratio d) calculated pattern from (**1**)(**12tfib**) single crystal data.	28
Figure S19.	PXRD patterns of: a) **1**, b) product obtained by grinding **13tfib** and **1** in a 1:2 stoichiometric ratio, c) calculated pattern from (**1**)(**13tfib**)$_2$ single crystal data.	28
Figure S20.	PXRD patterns of: a) **14tfib**, b) **1**, c) product obtained by grinding **14tfib** and **1** in a 1:2 stoichiometric ratio, d) calculated pattern from (**1**)(**14tfib**)$_2$ single crystal data.	29
Figure S21.	PXRD patterns of: a) **135tfib**, b) **1**, c) product obtained by grinding **135tfib** and **1** in a 1:2 stoichiometric ratio, d) calculated pattern from (**1**)(**135tfib**)$_2$ single crystal data.	29
Figure S22.	PXRD patterns of: a) **1**, b) product obtained by grinding **ipfb** and **1** in a 1:2 stoichiometric ratio, c) calculated pattern from (**1**)(**ipfb**)$_2$ single crystal data.	30
Figure S23.	PXRD patterns of: a) **12tfib**, b) **2**, c) product obtained by grinding **12tfib** and **2** in a 1:1 stoichiometric ratio, d) calculated pattern from (**1**)(**12tfib**)$_2$ single crystal data.	30
Figure S24.	PXRD patterns of: a) **2**, b) product obtained by grinding **13tfib** and **2** in a 2:3 stoichiometric ratio, c) calculated pattern from (**2**)$_2$(**13tfib**)$_3$ single crystal data.	31
Figure S25.	PXRD patterns of: a) **14tfib**, b) **2**, c) product obtained by grinding **14tfib** and **2** in a 1:2 stoichiometric ratio, d) calculated pattern from (**2**)(**14tfib**)$_2$ single crystal data.	31
Figure S26.	PXRD patterns of: a) **135tfib**, b) **2**, c) product obtained by grinding **135tfib** and **2** in a 1:1 stoichiometric ratio, d) calculated pattern from (**2**)(**135tfib**) single crystal data.	32
Figure S27.	PXRD patterns of: a) **2**, b) product obtained by grinding **ipfb** and **2** in a 1:2 stoichiometric ratio, c) calculated pattern from (**1**)(**ipfb**)$_2$ single crystal data.	32
Figure S28.	PXRD patterns of: a) **1**, b) product obtained by vapor sorption experiments of **ipfb** and **1** c) calculated pattern from (**1**)$_2$(**ipfb**)$_3$ single crystal data	33

Figure S29.	PXRD patterns of: a) **1**, b) product obtained by vapor sorption experiments of **13tfib** and **1** c) calculated pattern from (**1**)(**13tfib**)$_2$ single crystal data.	33
Figure S30.	TG curve of **1**.	34
Figure S31.	TG curve of **2**.	34
Figure S32.	TG curve of the cocrystal (**1**)(**12tfib**).	35
Figure S33.	TG curve of the cocrystal (**1**)(**13tfib**)$_2$.	35
Figure S34.	TG curve of the cocrystal (**1**)(**14tfib**)$_2$.	36
Figure S35.	TG curve of the cocrystal (**1**)(**135tfib**)$_2$.	36
Figure S36.	TG curve of the cocrystal (**1**)(**ipfb**)$_2$.	37
Figure S37.	TG curve of the cocrystal (**1**)(**ipfb**)$_2$.	37
Figure S38.	TG curve of the cocrystal (**2**)(**12tfib**).	38
Figure S39.	TG curve of the cocrystal (**2**)$_2$(**13tfib**)$_3$.	38
Figure S40.	TG curve of the cocrystal (**2**)$_2$(**14tfib**)$_3$.	39
Figure S41.	TG curve of the cocrystal (**2**)(**135tfib**).	39
Figure S42.	TG curve of the cocrystal (**2**)(**ipfb**)$_2$.	40
Figure S43.	TG curve of the cocrystal (**1**)$_2$(**14tfib**)$_3$(**ACN**)$_2$	40
Figure S44.	TG curve of the cocrystal (**1**)$_2$(**14tfib**)$_3$(**NMT**)$_2$	41
Figure S45.	TG curve of the cocrystal (**1**)$_2$(**14tfib**)$_3$(**ACT**)$_2$	41

MECHANOCHEMICAL SYNTHESES

Synthesis of (**1**)(**12tfib**)

A mixture of **1** (57.87 mg, 104 μmol) and **12tfib** (42.16 mg, 105 μmol) was placed in a 10 mL stainless steel jar along with 20 μL methanol and one stainless steel ball 12 mm in diameter. The reaction mixture was then milled for 30 minutes in a Retsch MM200 Shaker Mill operating at 25 Hz.

Synthesis of (**1**)(**13tfib**)$_2$

A mixture of **1** (28.48 mg, 51 μmol) and **13tfib** (15.6 μL, 104 μmol) was placed in a 10 mL stainless steel jar along with 20 μL methanol and two stainless steel balls 7 mm in diameter. The reaction mixture was then milled for 60 minutes in a Retsch MM200 Shaker Mill operating at 25 Hz.

Synthesis of (**1**)(**14tfib**)$_2$

A mixture of **1** (40.68 mg, 74 μmol) and **14tfib** (59.32 mg, 148 μmol) was placed in a 10 mL stainless steel jar along with 20 μL methanol and two stainless steel balls 7 mm in diameter. The reaction mixture was then milled for 60 minutes in a Retsch MM200 Shaker Mill operating at 25 Hz.

Synthesis of (**1**)(**135tfib**)$_2$

A mixture of **1** (35.10 mg, 64 μmol) and **135tfib** (64.90 mg, 128 μmol) was placed in a 10 mL stainless steel jar along with 20 μL methanol two stainless steel balls 7 mm in diameter. The reaction mixture was then milled for 60 minutes in a Retsch MM200 Shaker Mill operating at 25 Hz.

Synthesis of (**1**)$_2$(**ipfb**)$_3$

A mixture of **1** (48.40 mg, 87 μmol) and **ipfb** (23.50 μL, 176 μmol) was placed in a 10 mL stainless steel jar along with 20 μL methanole and one stainless steel ball 12 mm in diameter. The reaction mixture was then milled for 30 minutes in a Retsch MM200 Shaker Mill operating at 25 Hz.

Synthesis of (**2**)(**12tfib**)$_2$

A mixture of **2** (38.26 mg, 77 μmol) and **12tfib** (61.74 mg, 154 μmol) was placed in a 10 mL stainless steel jar along with 20 μL acetonitrile and one stainless steel ball 12 mm in diameter. The reaction mixture was then milled for 30 minutes in a Retsch MM200 Shaker Mill operating at 25 Hz.

Synthesis of (**2**)$_2$(**13tfib**)$_3$

A mixture of **2** (36.20 mg, 73 μmol) and **13tfib** (20 μL, 133 μmol) was placed in a 10 mL stainless steel jar along with 20 μL acetonitrile and one stainless steel ball 12 mm in diameter. The reaction mixture was then milled for 30 minutes in a Retsch MM200 Shaker Mill operating at 25 Hz.

Synthesis of (**2**)$_2$(**14tfib**)$_3$

A mixture of **2** (45.24 mg, 91 μmol) and **14tfib** (54.76 mg, 136 μmol) was placed in a 10 mL stainless steel jar along with 20 μL acetonitrile and one stainless steel ball 12 mm in diameter. The reaction mixture was then milled for 30 minutes in a Retsch MM200 Shaker Mill operating at 25 Hz.

Synthesis of (**2**)(**135tfib**)

A mixture of **2** (49.42 mg, 99 μmol) and **135tfib** (50.58 mg, 99 μmol) was placed in a 10 mL stainless steel jar along with 20 μL acetonitrile and one stainless steel ball 12 mm in diameter. The reaction mixture was then milled for 30 minutes in a Retsch MM200 Shaker Mill operating at 25 Hz.

Synthesis of (**2**)(**ipfb**)$_2$

A mixture of **2** (45.86 mg, 92 μmol) and **ipfb** (24.6 μL, 185 μmol) was placed in a 10 mL stainless steel jar along with 20 μL acetonitrile and one stainless steel ball 12 mm in diameter. The reaction mixture was then milled for 30 minutes in a Retsch MM200 Shaker Mill operating at 25 Hz.

Synthesis of (**1**)$_2$(**14tfib**)$_3$(**ACN**)$_2$

A mixture of **1** (40.68 mg, 74 μmol) and **14tfib** (44.60 mg, 111 μmol) was placed in a 10 mL stainless steel jar along with 20 μL acetonitrile and two stainless steel balls 7 mm in diameter. The reaction mixture was then milled for 60 minutes in a Retsch MM200 Shaker Mill operating at 25 Hz.

Synthesis of (**1**)$_2$(**14tfib**)$_3$(**NMT**)$_2$

A mixture of **1** (40.68 mg, 74 μmol) and **14tfib** (44.60 mg, 111 μmol) was placed in a 10 mL stainless steel jar along with 20 μL nitrometane and two stainless steel balls 7 mm in diameter. The reaction mixture was then milled for 60 minutes in a Retsch MM200 Shaker Mill operating at 25 Hz.

SOLUTION SYNTHESES

Single crystals of (**1**)(**12tfib**)

A mixture of **1** (12.57 mg, 23 μmol) and **12tfib** (10 mg, 25 μmol) was dissolved in 2.0 of hot chloroform. After dissolving, 3.0 mL of methanol was added into the solution and left to crystallize at room temperature.

Single crystals of (**1**)(**13tfib**)$_2$

A mixture of **1** (10 mg, 18 μmol) and **13tfib** (6 μL, 36 μmol) was dissolved in 2.0 mL of hot dioxane and left to crystallize at room temperature.

Single crystals of (**1**)(**14tfib**)$_2$

A mixture of **1** (10 mg, 18 μmol) and **14tfib** (14.60 mg, 36 μmol) was dissolved in 2.0 mL of hot dioxane and left to crystallize at room temperature.

Single crystals of (**1**)(**135tfib**)$_2$

A mixture of **1** (10 mg, 18 μmol) and **135tfib** (18.60 mg, 36 μmol) was dissolved in 2.0 mL of hot methanol and left to crystallize at room temperature.

Single crystals of **(1)(ipfb)**$_2$

A mixture of **1** (13.05 mg, 24 μmol) and **ipfb** (8 μL, 60 μmol) was dissolved in 4.0 mL of hot methanol and left to crystallize at room temperature.

Single crystals of **(2)(12tfib)**$_2$

A mixture of **2** (10 mg, 20 μmol) and **12tfib** (16.07 mg, 40 μmol) was dissolved in 2.0 mL of hot chloroform. After dissolving, 3.0 mL of methanol was added into the solution and left to crystallize at room temperature and left to crystallize at room temperature.

Single crystals of **(2)**$_2$**(13tfib)**$_3$

A mixture of **2** (10 mg, 20 μmol) and **13tfib** (7 μL, 42 μmol) was dissolved in 2.0 mL of hot methanol mixed with 600 μL of water and left to crystallize at room temperature.

Single crystals of **(2)**$_2$**(14tfib)**$_3$

A mixture of **2** (10 mg, 20 μmol) and **14tfib** (16.40 mg, 40 μmol) was dissolved in 1.0 mL of hot methanol and left to crystallize at room temperature.

Single crystals of **(2)(135tfib)**$_2$

A mixture of **2** (10 mg, 20 μmol) and **135tfib** (20.80 mg, 40 μmol) was dissolved in 3.0 mL of hot methanol mixed with 400 μL of water and left to crystallize at room temperature.

Single crystals of **(2)(ipfb)**$_2$

A mixture of **2** (12.57 mg, 25 μmol) and **ipfb** (8 μL, 60 μmol) was dissolved in 3.0 mL of hot methanol and left to crystallize at room temperature.

Single crystals of **(1)**$_2$**(ipfb)**$_3$

A mixture of **1** (13.05 mg, 24 μmol) and **ipfb** (5 μL, 37 μmol) was dissolved in 4.0 mL of hot methanol. After dissolving, 2.0 mL of acetone was added into the solution and left to crystallize at room temperature.

Single crystals of **(1)**$_2$**(14tfib)**$_3$**(ACT)**$_2$

A mixture of **1** (10 mg, 18 μmol) and **14tfib** (14.60 mg, 36 μmol) was dissolved in 4.0 mL of hot acetone and left to crystallize at room temperature.

Single crystals of **(1)**$_2$**(14tfib)**$_3$**(ACN)**$_2$

A mixture of **1** (10 mg, 18 μmol) and **14tfib** (14.60 mg, 36 μmol) was dissolved in 4.0 mL of hot acetonitrile and left to crystallize at room temperature.

Single crystals of $(\mathbf{1})_2(\mathbf{14tfib})_3(\mathbf{NMT})_2$

A mixture of **1** (10 mg, 18 μmol) and **14tfib** (14.60 mg, 36 μmol) was dissolved in 4.0 mL of hot nitrometane and left to crystallize at room temperature.

THERMAL ANALYSIS

TGA measurements were performed on a Mettler-Toledo TGA/DSC 3+ module. The samples were placed in open 70 μL alumina pans, and heated from 30 °C to 800 °C for coordination compounds **1**, **2**, and prepared cocrystals at a rate of 10 °C min^{-1} under nitrogen flow of 50 mL min^{-1}. The data collection and analysis were performed using the program package STARe Software 15.00.[1]

POWDER X-RAY DIFFRACTION EXPERIMENTS

PXRD experiments were performed on a Malvern PANalytical *Aeris* X-ray diffractometer with Cu$K_{\alpha 1}$ (1.54056 Å) radiation at 15 mA and 40 kV. The scattered intensities were measured with a line (1D) detector. The angular range was from 5 to 40° (2θ) with an interpolated step size of 0.00543322°. Data analysis was performed using the program *Data Viewer*.[2]

SINGLE-CRYSTAL X-RAY DIFFRACTION EXPERIMENTS

The crystal and molecular structures of the prepared cocrystals were determined by single-crystal X-ray diffraction. Details of data collection and crystal structure refinement are listed in Table S1. Diffraction measurements were made on and Rigaku Synergy XtaLAB X-ray diffractometer with graphite-monochromated MoK_{α} (λ = 0.71073Å) radiation. The data sets were collected using the ω scan mode over the 2θ range up to 64°. The *CrysAlisPro* program package was employed for data collection, cell refinement, and data reduction.[3] The structures were solved by direct methods and refined using the *SHELXS*, *SHELXT*, and *SHELXL* programs, respectively.[4,5] The structural refinement was performed on F^2 using all data. Hydrogen atoms were placed in calculated positions and treated as riding on their parent atoms All calculations were performed using the *WINGX* crystallographic suite of programs.[6]

The molecular structures of compounds and their molecular packing projections were prepared by *Mercury*.[7]

References

1. STARe Evaluation Software Version 15.00, Mettler–Toledo GmbH, 2016.
2. Data Viewer Version 1.9a, PANalytical B.V. Amelo, The Netherlands, 2018.
3. Rigaku Oxford Diffraction, Gemini CCD system , CrysAlis Pro software, Version 171.41.93a, 2020.
4. G. M. Sheldrick, *Acta Cryst. A*, 2015, **71**, 3–8.
5. (a) G. M. Sheldrick, *Acta Cryst. A*, 2008, **64**, 112–122; (b) G. M. Sheldrick, *Acta Cryst. C*, 2015, **71**, 3–8.
6. L. J. Farrugia, *J. Appl. Cryst.*, 2012, **45**, 849–854.
7. C. F. Macrae, I. J. Bruno, J. A. Chisholm, P. R. Edgington, P. McCabe, E. Pidcock, L. Rodriguez-Monge, R. Taylor, J. v. d. Streek and P. A. Wood, *J. Appl. Crystallogr.* **2008**, 41, 466

Table S1. Crystal data and refinement details for the prepared compounds.

	**(1)(12tfib)**	**(1)(13tfib)$_2$**
Molecular formula	$(C_{26}H_{28}N_6S_2Ni)(C_6F_4I_2)$	$(C_{26}H_{28}N_6S_2Ni)(C_6F_4I_2)_2$
M_r	949.23	1351.09
Crystal system	monoclinic	orthorhombic
Space group	*I2/a*	*Pccn*
Crystal data:		
a / Å	16.6553(7)	14.9090(8)
b / Å	17.2744(7)	18.3951(9)
c / Å	27.4413(10)	16.9356(8)
α / °	90	90
β / °	106.074(4)	90
γ / °	90	90
V / Å^3	7586.5(5)	4644.6(4)
Z	8	4
D_{calc} / g cm^{-3}	1.662	1.932
λ(MoK_α) / Å	0.71073	0.71073
T / K	295	295
Crystal size / mm^3	0.40 x 0.25 x 0.2	0.48 x 0.31 x 0.1
μ / mm^{-1}	2.299	3.233
F(000)	3712	2568
Refl. collected/unique	50085 / 12718	35235/ 7823
Parameters/restraints	428 / 0	269/ 0
$\Delta\rho_{max}$, $\Delta\rho_{min}$ / e Å^{-3}	0.871; -0.534	3.573; -2.546
$R[F^2 > 4\sigma(F^2)]$	0.0345	0.0696
w$R(F^2)$	0.0852	0.2032
Goodness-of-fit, S	1.050	1.044
CCDC deposition number	2354516	2354514

Table S1. continued

	**(1)(14tfib)$_2$**	**(1)(135tfib)$_2$**
Molecular formula	$(C_{26}H_{28}N_6S_2Ni)(C_6F_4I_2)_2$	$(C_{26}H_{28}N_6S_2Ni)(C_6F_4I_2)_2$
M_r	1351.09	1566.89
Crystal system	triclinic	monoclinic
Space group	*P–1*	*P2$_1$/n*
Crystal data:		
a / Å	9.8344(3)	17.8895(19)
b / Å	10.5674(4)	13.9062(8)
c / Å	11.9171(4)	20.8113(11)
α / °	98.059(3)	90
β / °	107.691(3)	107.672(8)
γ / °	94.734(3)	90
V / Å^3	1157.93(7)	4933.0(7)
Z	1	4
D_{calc} / g cm^{-3}	1.938	2.110
λ(MoK_α) / Å	0.71073	0.71073
T / K	295	170
Crystal size / mm^3	0.44 x 0.30 x 0.21	0.49x 0.33 x 0.13
μ / mm^{-1}	3.242	4.289
F(000)	642	2920
Refl. collected/unique	25013/ 7823	66672/ 16477
Parameters/restraints	270/ 0	536/ 0
$\Delta\rho_{max}$, $\Delta\rho_{min}$ / e Å^{-3}	1.315; -0.959	1.147; -1.299
$R[F^2 > 4\sigma(F^2)]$	0.0402	0.0435
w$R(F^2)$	0.1090	0.1372
Goodness-of-fit, S	1.062	1.141
CCDC deposition number	2354512	2354520

Table S1. continued

	**(1)(ipfb)$_2$**	**(2)(12tfib)$_2$**
Molecular formula	$(C_{26}H_{28}N_6S_2Ni)(C_6F_5I)_2$	$(C_{22}H_{20}N_6S_2Ni)(C_6F_4I_2)_2$
M_r	1135.29	1294.99
Crystal system	orthorhombic	monoclinic
Space group	*Pccn*	$P2_1/c$
Crystal data:		
a / Å	14.8446(7)	23.0898(4)
b / Å	18.6509(10)	15.6231(3)
c / Å	16.5380(8)	23.3866(4)
α / °	90	90
β / °	90	90.0240(10)
γ / °	90	90
V / Å^3	4578.8(4)	8436.3(3)
Z	4	8
D_{calc} / g cm^{-3}	1.647	2.039
$\lambda(MoK_\alpha)$ / Å	0.71073	0.71073
T / K	295	293
Crystal size / mm^3	0.48 x 0.37 x 0.27	0.47x 0.44 x 0.20
μ / mm^{-1}	1.938	3.555
$F(000)$	2216	4880
Refl. collected/unique	32010/ 7696	246481/ 28878
Parameters/restraints	269/ 0	991/ 0
$\Delta\rho_{max}$, $\Delta\rho_{min}$ / e Å^{-3}	1.086; -1.079	1.242; -1.351
$R[F^2 > 4\sigma(F^2)]$	0.0582	0.0694
$wR(F^2)$	0.1868	0.1817
Goodness-of-fit, S	1.070	1.100
CCDC deposition number	2354513	2354525

Table S1. continued

	**(2)$_2$(13tfib)$_3$**	**(2)$_2$(14tfib)$_3$**
Molecular formula	$(C_{22}H_{20}N_6S_2Ni)_2(C_6F_5I_2)_3$	$(C_{22}H_{20}N_6S_2Ni)_2(C_6F_5I_2)_3$
M_r	2188.12	1094.06
Crystal system	triclinic	monoclinic
Space group	*P–1*	$P2_1/c$
Crystal data:		
a / Å	12.4614(3)	10.3526(4)
b / Å	15.9741(3)	12.8846(5)
c / Å	20.4845(5)	28.9055(10)
α / °	91.881(2)	90
β / °	96.469(2)	96.679(3)
γ / °	112.583(2)	90
V / Å^3	3728.12(15)	3829.5(2)
Z	2	2
D_{calc} / g cm^{-3}	1.949	1.898
λ(MoK_α) / Å	0.71073	0.71073
T / K	295	295
Crystal size / mm^3	0.44 x 0.32 x 0.15	0.31 x 0.26 x 0.11
μ / mm^{-1}	3.178	3.094
F(000)	2084	2084
Refl. collected/unique	147192/ 25545	51741 / 13081
Parameters/restraints	883/ 0	442 / 0
$\Delta\rho_{max}$, $\Delta\rho_{min}$ / e Å^{-3}	3.510; -1.299	0.981; -1.491
$R[F^2 > 4\sigma(F^2)]$	0.0998	0.0470
w$R(F^2)$	0.2726	0.1439
Goodness-of-fit, S	1.116	1.034
CCDC deposition number	2354524	2354521

Table S1. continued

	**(2)(135tfib)**	**(2)(ipfb)$_2$**
Molecular formula	$(C_{22}H_{20}N_6S_2Ni)(C_6F_3I_3)$	$(C_{22}H_{20}N_6S_2Ni)(C_6F_5I_1)_2$
M_r	1001.03	1079.19
Crystal system	monoclinic	orthorombic
Space group	$P2_1/n$	$Pnna$
Crystal data:		
a / Å	9.6263(4)	15.9678(5)
b / Å	25.3845(12)	26.2901(8)
c / Å	13.7817(7)	9.6727(3)
α / °	90	90
β / °	94.250(4)	90
γ / °	90	90
V / Å^3	3358.4(3)	4060.6(2)
Z	4	4
D_{calc} / g cm^{-3}	1.980	1.765
$\lambda(MoK_\alpha)$ / Å	0.71073	0.71073
T / K	295	295
Crystal size / mm^3	0.49 x 0.26 x 0.12	0.30 x 0.20 x 0.19
μ / mm^{-1}	3.505	2.180
F(000)	1904	2088
Refl. collected/unique	37921 / 9785	39258/ 6881
Parameters/restraints	388 / 0	252 / 0
$\Delta\rho_{max}$, $\Delta\rho_{min}$ / e Å^{-3}	1.716; -1.921	0.821; -0.723
$R[F^2 > 4\sigma(F^2)]$	0.0707	0.0355
w$R(F^2)$	0.1724	0.0976
Goodness-of-fit, S	1.050	1.043
CCDC deposition number	2354518	2354515

Table S1. continued

	**(1)$_2$(ipfb)$_3$**	**(1)$_2$(14tfib)$_3$(ACT)$_2$**
Molecular formula	$(C_{26}H_{28}N_6S_2Ni)_2$ $(C_6F_5I)_3$	$(C_{26}H_{28}N_6S_2Ni)_2(C_6F_4I_2)_3$ $(C_3H_6O)_2$
M_r	1976.62	1208.24
Crystal system	triclinic	monoclinic
Space group	P–1	$P2_1/c$
Crystal data:		
a / Å	14.2683(2)	11.4323(4)
b / Å	14.7546(3)	17.7800(5)
c / Å	21.7239(3)	22.8990(7)
α / °	91.1330(10)	90
β / °	102.6490(10)	94.899(3)
γ / °	111.747(2)	90
V / Å^3	4119.02(13)	4637.6(3)
Z	2	4
D_{calc} / g cm^{-3}	1.594	1.730
$\lambda(MoK_\alpha)$ / Å	0.71073	0.71073
T / K	293	293
Crystal size / mm^3	0.42 x 0.35 x 0.33	0.35 x 0.29 x 0.11
μ / mm^{-1}	1.763	2.565
$F(000)$	1948	2340
Refl. collected/unique	164017/ 28263	63905/ 15594
Parameters/restraints	963/ 0	516/ 1
$\Delta\rho_{max}$, $\Delta\rho_{min}$ / e Å^{-3}	1.328; -1.091	1.188; -0.725
$R[F^2 > 4\sigma(F^2)]$	0.0438	0.0356
w$R(F^2)$	0.1339	0.0937
Goodness-of-fit, S	1.047	1.040
CCDC deposition number	2354523	2354519

Table S1. continued

	**(1)$_2$(14tfib)$_3$(ACN)$_2$**	**(1)$_2$(14tfib)$_3$(NMT)$_2$**
Molecular formula	$(C_{26}H_{28}N_6S_2Ni)_2(C_6F_4I)_3$ $(C_2H_3N)_2$	$(C_{26}H_{28}N_6S_2Ni)_{2(}C_6F_4I_2)_3$ $(CH_3NO_2)_2$
M_r	1191.22	1211.21
Crystal system	monoclinic	monoclinic
Space group	$P2_1/c$	$P2_1/c$
Crystal data:		
a / Å	11.3988(4)	11.4202(3)
b / Å	17.5672(6)	17.7131(5)
c / Å	22.5234(8)	22.4475(8)
α / °	90	90
β / °	95.110(3)	95.732(3)
γ / °	90	90
V / Å^3	4492.3(3)	4518.1(2)
Z	4	4
D_{calc} / g cm^{-3}	1.761	1.781
λ(MoK_α) / Å	0.71073	0.71073
T / K	295	293
Crystal size / mm^3	0.49 x 0.36 x 0.15	0.41 x 0.21 x 0.15
μ / mm^{-1}	2.646	2.635
F(000)	2300	2340
Refl. collected/unique	49587 / 14983	50513/ 13169
Parameters/restraints	511 / 0	519 / 0
$\Delta\rho_{max}$, $\Delta\rho_{min}$ / e Å^{-3}	1.272; -1.529	0.924; -0.981
$R[F^2 > 4\sigma(F^2)]$	0.0543	0.0363
w$R(F^2)$	0.1737	0.0946
Goodness-of-fit, S	1.033	1.058
CCDC deposition number	2354517	2354522

Table S2. Rotation angles (∠) of isothiocyanate group (Ni-NCS) present in the herein prepared cocrystals and starting coordination compound **1**.

Cocrystal	Ni-N-C	∠ (Ni-N-C)/°
**(1)(12tfib)**	Ni1-N1-C1	165.13
	Ni1-N4-C2	159.19
**(1)(13tfib)**$_2$	Ni1-N1-C1	137.60
**(1)(14tfib)**$_2$	Ni1-N1-C1	164.51
**(1)**$_2$**(14tfib)**$_3$**(ACT)**$_2$	Ni1-N1-C1	172.16
	Ni1-N2-C2	162.75
**(1)**$_2$**(14tfib)**$_3$**(NMT)**$_2$	Ni1-N3-C36	155.66
	Ni1-N6-C35	176.24
**(1)**$_2$**(14tfib)**$_3$**(ACN)**$_2$	Ni1-N1-C34	176.97
	Ni1-N1-C35	155.21
**(1)(135tfib)**$_2$	Ni1-N1-C1	173.60
**(1)(ipfb)**$_2$	Ni1-N1-C1	172.59
**(1)**$_2$**(ipfb)**$_3$	Ni1-N1-C19	172.59
	Ni1-N1-C20	177.07
	Ni2-N8-C22	168.33
	Ni2-N11-C21	172.31
**1**	Ni-N-C	155.13

Table S3. Rotation angles (∠) of isothiocyanate group (Ni-NCS) present in the herein prepared cocrystals and starting coordination compound **2**.

Cocrystal	Ni-N-C	∠ (Ni-N-C)/°
**(2)(12tfib)**$_2$	Ni1-N1-C3	160.15
	Ni1-N4-C4	174.04
	Ni2-N7-C1	170.87
	Ni2-N10-C2	170.70
**(2)**$_2$**(13tfib)**$_3$	Ni1-N3-C39	150.86
	Ni1-N07-C40	164.29
	Ni07-N6-C42	155.44
	Ni07-N9-C41	155.37
**(2)**$_2$**(14tfib)**$_3$	Ni1-N1-C1	173.89
**(2)(135tfib)**$_2$	Ni1-N1-C1	159.16
	Ni1-N3-C2	152.36
**(2)(ipfb)**$_2$	Ni1-N4-C1	168.56
	Ni1-N4-C1	168.56
**2**	Ni-N-C	156.90

Table S4. Rotation angles (∠) of pyridine rings present in the herein prepared cocrystals and starting coordination compound **1**: A) rotation angles of planes planar with pyridine rings (py) and plane containing nickel atom and four nitrogen atoms (from pyridine ligands), B) rotation angles of two opposite pyridine rings.

		**A**		**B**
Cocrystal	plane(Ni)-py(N)	∠ plane(Ni)-py(N) / °	py(N)/py(N)	∠ py(N)/py(N) / °
**(1)(12tfib)**	plane(Ni)- py(N2)	54.17	py(N2)/py(N5)	76.21
	plane(Ni)- py(N3)	51.30	py(N3)/ py(N6)	76.53
	plane (Ni)- py(N5)	49.62		
	plane(Ni)- py(N6)	52.21		
**(1)(13tfib)**$_2$	plane(Ni)- py(N2)	51.16	py(N2)/ py(N3)	78.76
	plane(Ni)- py(N3)	51.32	py(N2)/ py(N3)	78.76
**(1)(14tfib)**$_2$	plane(Ni)- py(N2)	49.88	py(N3)/ py(N3)	0
	plane(Ni)- py(N3)	77.33	py(N2)/ py(N2)	0
**(1)**$_2$**(14tfib)**$_3$**(ACT)**$_2$	plane(Ni)- py(N3)	58.33	py(N3)/ py(N5)	56.58
	plane(Ni)- py(N4)	50.88	py(N4)/ py(N6)	82.91
	plane(Ni)- py(N5)	66.29		
	plane(Ni)- py(N6)	46.53		
**(1)**$_2$**(14tfib)**$_3$**(NMT)**$_2$	plane(Ni)- py(N1)	48.96	py(N1)/ py(N5)	71.13
	plane(Ni)- py(N2)	53.60	py(N2)/ py(N4)	68.94
	plane(Ni)- py(N4)	57.76		
	plane(Ni)- py(N5)	60.63		
**(1)**$_2$**(14tfib)**$_3$**(ACN)**$_2$	plane(Ni)- py(N3)	60.82	py(N3)/ py(N5)	72.06
	plane(Ni)- py(N4)	52.49	py(N4)/ py(N6)	70.12
	plane(Ni)- py(N5)	47.93		
	plane(Ni)- py(N6)	57.76		
**(1)(135tfib)**$_2$	plane(Ni)- py(N1)	49.77	py(N1)/ py(N3)	56.74
	plane(Ni)- py(N2)	62.81	py(N2)/ py(N4)	81.43
	plane(Ni)- py(N3)	73.74		
	plane(Ni)- py(N4)	35.83		
**(1)(ipfb)**$_2$	plane(Ni)- py(N2)	50.70	py(N2)/ py(N3)	79.40
	plane(Ni)- py(N3)	50.96	py(N2)/ py(N3)	79.40
**(1)**$_2$**(ipfb)**$_3$	plane(Ni1)- py(N2)	51.58	py(N2)/ py(N3)	74.99
	plane(Ni1)- py(N3)	53.87	py(N4)/ py(N6)	84.77
	plane(Ni1)- py(N4)	47.71		
	plane(Ni1)- py(N6)	47.52		
	plane(Ni2)- py(N7)	54.29	py(N7)/ py(N9)	72.57
	plane(Ni2)- py(N9)	53.14	py(N10)/ py(N12)	76.18
	plane(Ni2)- py(N10)	49.69		
	plane(Ni2)- py(N12)	54.17		
**1**	plane(Ni)- py(N1)	47.76	py(N1)/ py(N3)	73.73
	plane(Ni)- py(N3)	56.58	py(N1)/ py(N3)	73.73

Table S5. Rotation angles (∠) of pyridine rings present in the herein prepared cocrystals and starting coordination compound **2**: A) rotation angles of planes planar with pyridine rings (py) and plane containing nickel atom and four nitrogen atoms (from pyridine ligands), B) rotation angles of two opposite pyridine rings.

		**A**		**B**
Cocrystal	plane(Ni)-py(N)	∠ plane(Ni)-py(N) / °	py(N)/py(N)	∠ py(N)/py(N) / °
**(2)(12tfib)$_2$**	plane(Ni1)-N2(py)	50.36	py(N2)/ py(N5)	80.46
	plane(Ni1)-N3(py)	60.87	py(N3)/ py(N6)	73.83
	plane(Ni1)-N5(py)	50.20		
	plane(Ni1)-N6(py)	45.79		
	plane(Ni2)-N8(py)	48.89	py(N8)/ py(N11)	81.50
	plane(Ni2)-N9(py)	56.47	py(N9)/ py(N12)	73.34
	plane(Ni2)-N11(py)	73.83		
	plane(Ni2)-N12(py)	50.47		
**(2)$_2$(13tfib)$_3$**	plane(Ni)-N1(py)	57.28	py(N1)/ py(N4)	75.85
	plane(Ni)-N2(py)	51.14	py(N2)/ py(N5)	76.01
	plane(Ni)-N4(py)	47.03		
	plane(Ni)-N5(py)	52.87		
	plane(Ni07)-N7(py)	50.86	py(N7)/ py(N11)	73.66
	plane(Ni07)-N8(py)	53.07	py(N8)/ py(N10)	77.43
	plane(Ni07)-N10(py)	50.54		
	plane(Ni07)-N11(py)	55.84		
**(2)$_2$(14tfib)$_3$**	plane(Ni)-N1(py)	55.81	py(N1)/ py(N4)	66.23
	plane(Ni)-N3(py)	44.82	py(N3)/ py(N6)	86.91
	plane(Ni)-N4(py)	57.97		
	plane(Ni)-N6(py)	48.63		
**(2)(135tfib)$_2$**	plane(Ni)-N2(py)	51.32	py(N2)/ py(N4)	71.84
	plane(Ni)-N4(py)	60.02	py(N5)/ py(N6)	77.29
	plane(Ni)-N5(py)	59.77		
	plane(Ni)-N6(py)	42.94		
**(2)(ipfb)$_2$**	plane(Ni)-N1(py)	44.83	py(N1)/ py(N3)	87.12
	plane(Ni)-N2(py)	54.76	py(N2)/ py(N2)	70.74
	plane(Ni)-N3(py)	48.05		
**2**	plane(Ni)-N1(py)	57.14	py(N1)/ py(N1E)	0
	plane(Ni)-N2(py)	65.04	py(N2)/ py(N2E)	0
	plane(Ni)-N1E(py)	57.14		
	plane(Ni)-N2E(py)	65.04		

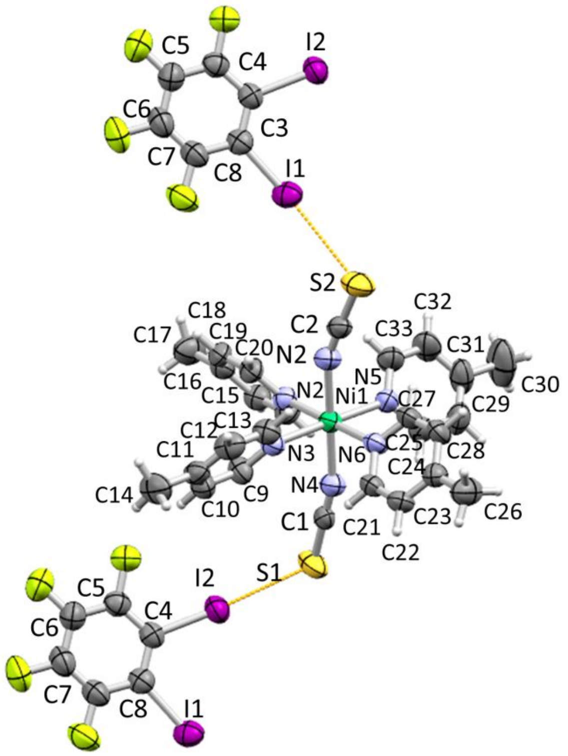

Figure S1. Molecular structure of (**1**)(**12tfib**) showing the atom-labeling scheme. Displacement ellipsoids are drawn at the 50 % probability level, halogen bonds are marked with orange dashed lines and H atoms are shown as small spheres of arbitrary radius.

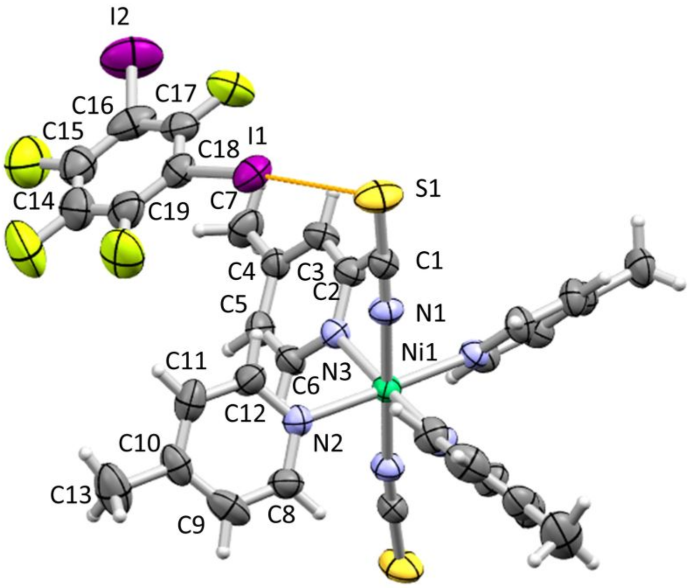

Figure S2. Molecular structure of (**1**)(**13tfib**)$_2$ showing the atom-labeling scheme. Displacement ellipsoids are drawn at the 50 % probability level, halogen bonds are marked with orange dashed lines and H atoms are shown as small spheres of arbitrary radius.

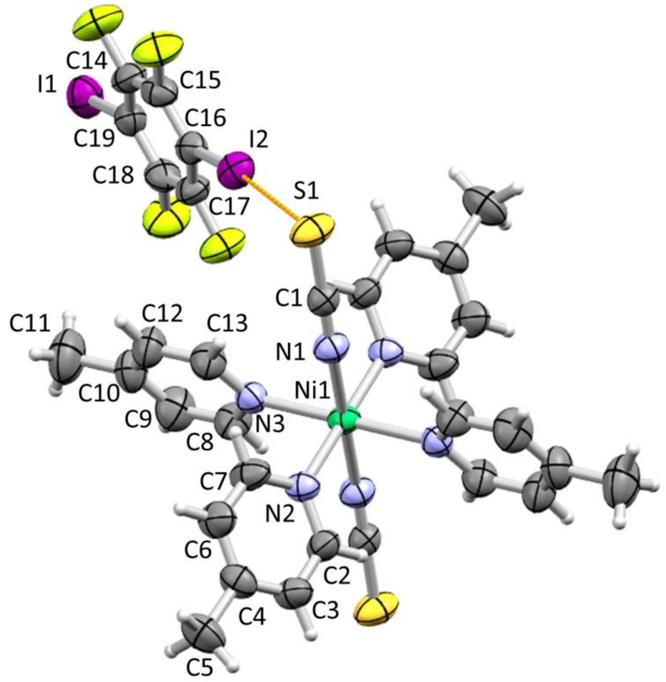

Figure S3. Molecular structure of (**1**)(**14tfib**)$_2$ showing the atom-labeling scheme. Displacement ellipsoids are drawn at the 50 % probability level, halogen bonds are marked with orange dashed lines and H atoms are shown as small spheres of arbitrary radius.

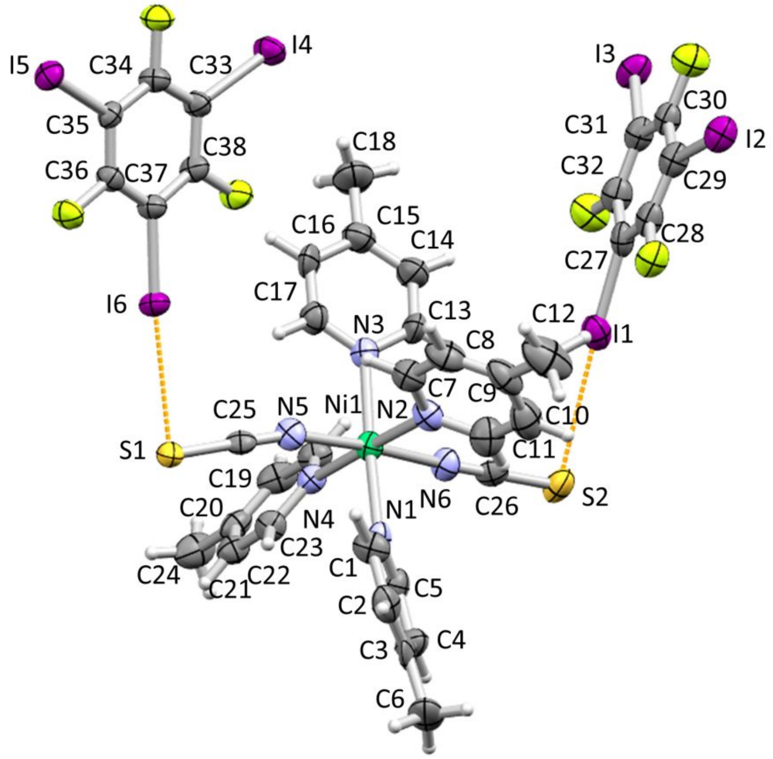

Figure S4. Molecular structure of (**1**)(**135tfib**)$_2$ showing the atom-labeling scheme. Displacement ellipsoids are drawn at the 50 % probability level, halogen bonds are marked with orange dashed lines and H atoms are shown as small spheres of arbitrary radius.

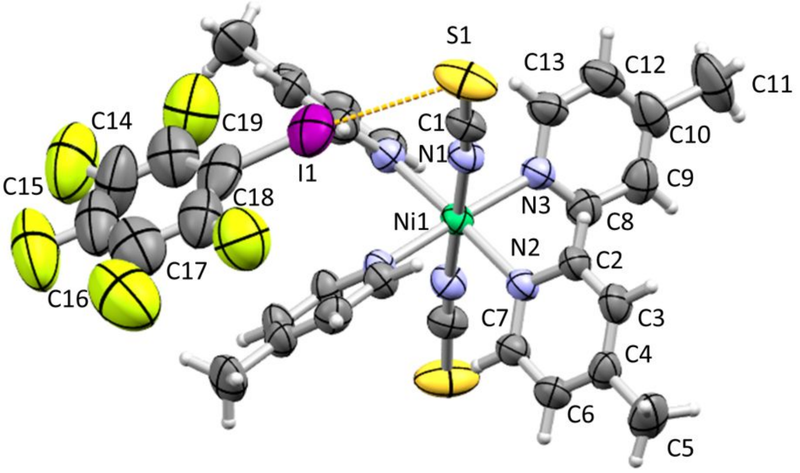

Figure S5. Molecular structure of (**1**)(**ipfb**)$_2$ showing the atom-labeling scheme. Displacement ellipsoids are drawn at the 50 % probability level, halogen bonds are marked with orange dashed lines and H atoms are shown as small spheres of arbitrary radius.

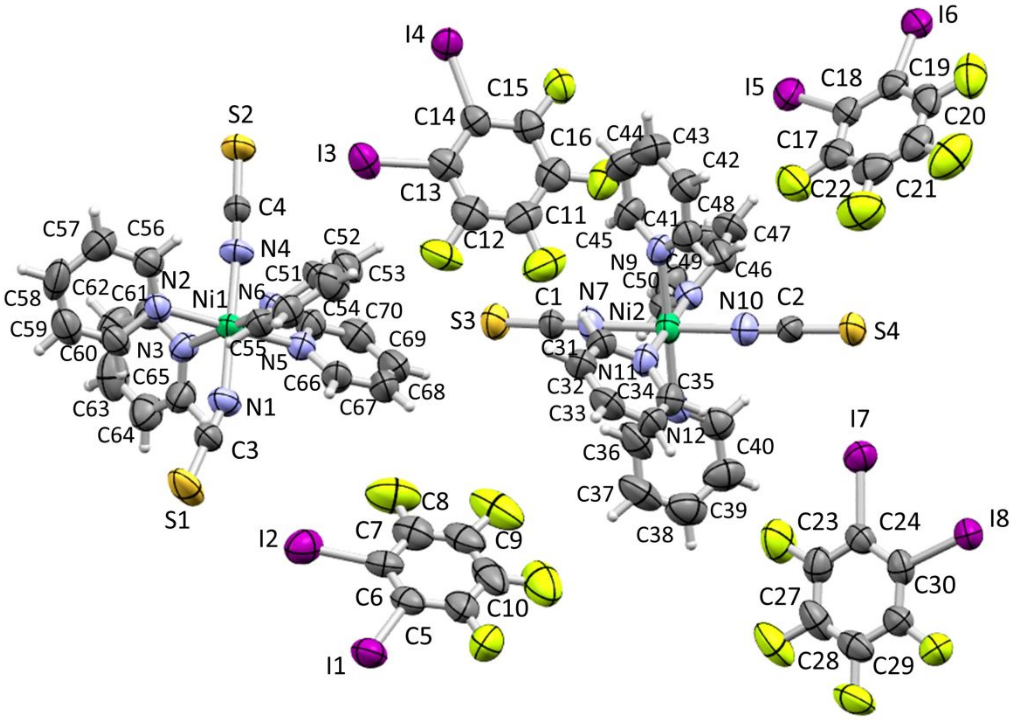

Figure S6. Molecular structure of (**2**)(**12tfib**)$_2$ showing the atom-labeling scheme. Displacement ellipsoids are drawn at the 50 % probability level and H atoms are shown as small spheres of arbitrary radius.

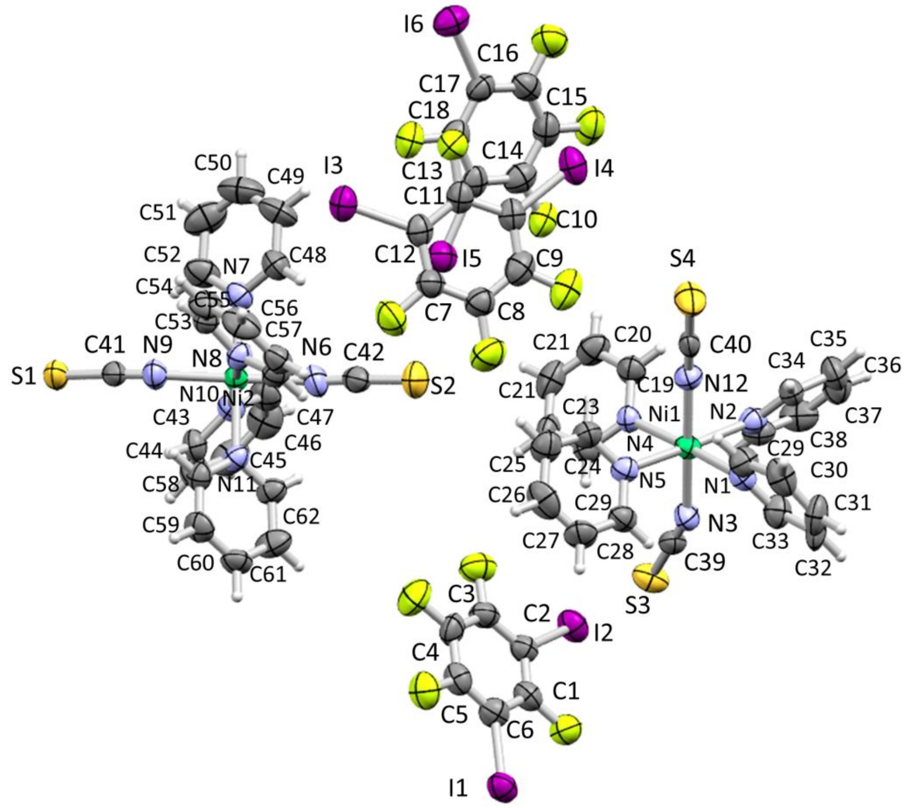

Figure S7. Molecular structure of (**2**)$_2$(**13tfib**)$_3$ showing the atom-labeling scheme. Displacement ellipsoids are drawn at the 50 % probability level and H atoms are shown as small spheres of arbitrary radius.

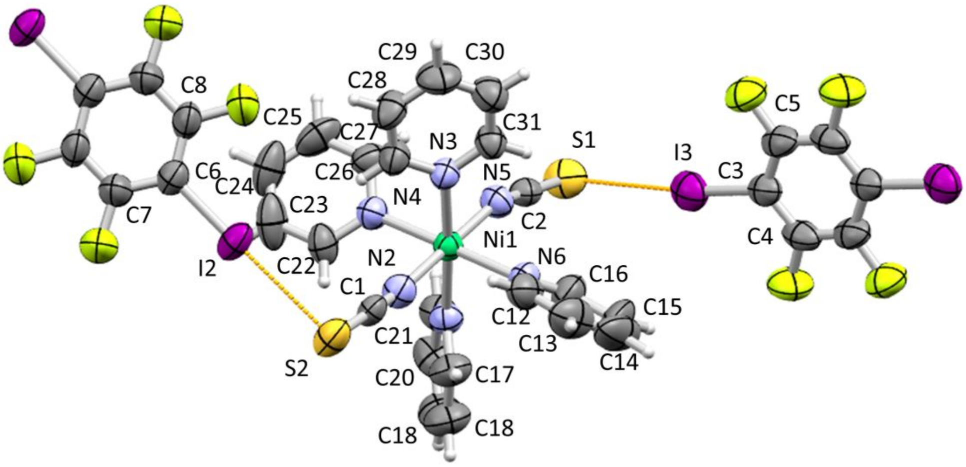

Figure S8. Molecular structure of (**2**)$_2$(**14tfib**)$_3$ showing the atom-labeling scheme. Displacement ellipsoids are drawn at the 50 % probability level, halogen bonds are marked with orange dashed lines and H atoms are shown as small spheres of arbitrary radius.

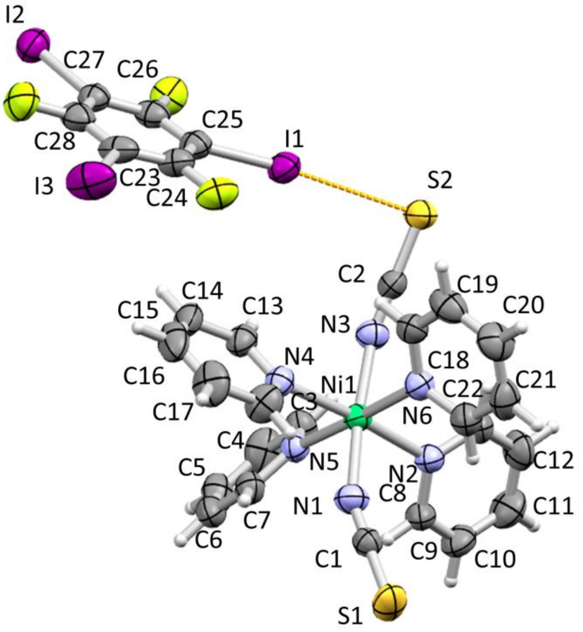

Figure S9. Molecular structure of (**2**)(**135tfib**) showing the atom-labeling scheme. Displacement ellipsoids are drawn at the 50 % probability level, halogen bonds are marked with orange dashed lines and H atoms are shown as small spheres of arbitrary radius.

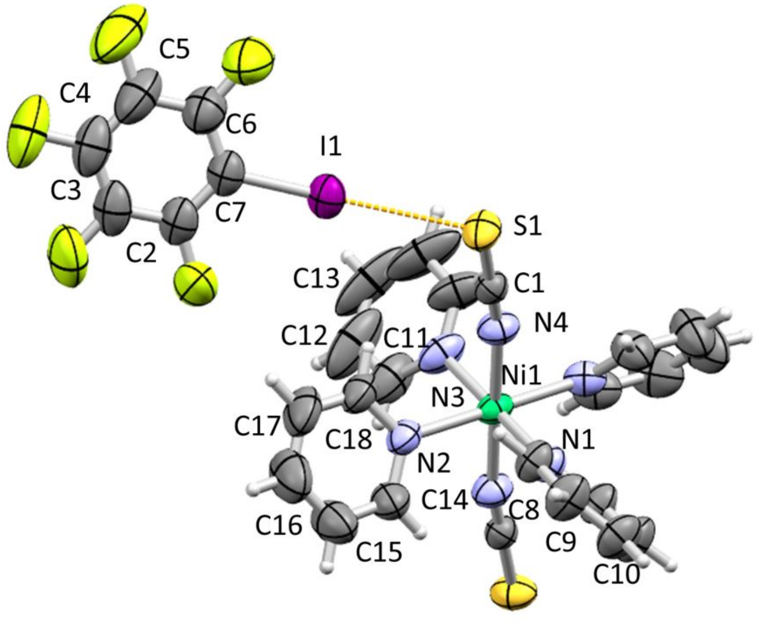

Figure S10. Molecular structure of (**2**)(**ipfb**)$_2$ showing the atom-labeling scheme. Displacement ellipsoids are drawn at the 50 % probability level, halogen bonds are marked with orange dashed lines and H atoms are shown as small spheres of arbitrary radius.

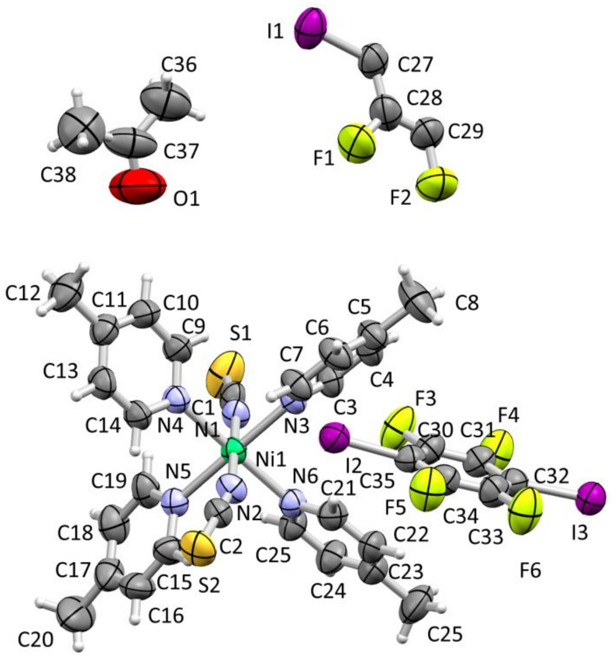

Figure S11. Molecular structure of (**1**)$_2$(**14tfib**)$_3$(**ACT**)$_2$ showing the atom-labeling scheme. Displacement ellipsoids are drawn at the 50 % probability level and H atoms are shown as small spheres of arbitrary radius.

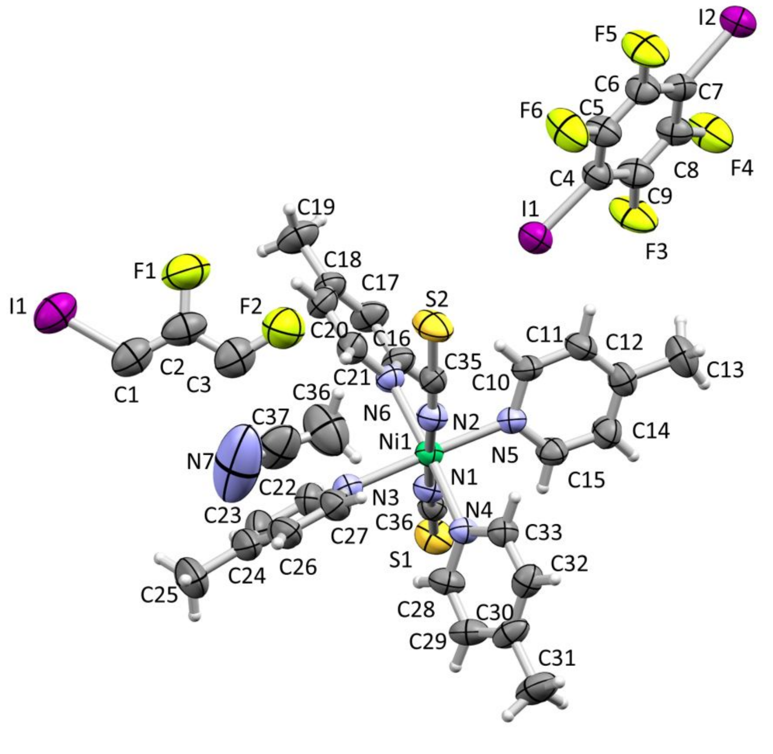

Figure S12. Molecular structure of (**1**)$_2$(**14tfib**)$_3$(**ACN**)$_2$ showing the atom-labeling scheme. Displacement ellipsoids are drawn at the 50 % probability level and H atoms are shown as small spheres of arbitrary radius.

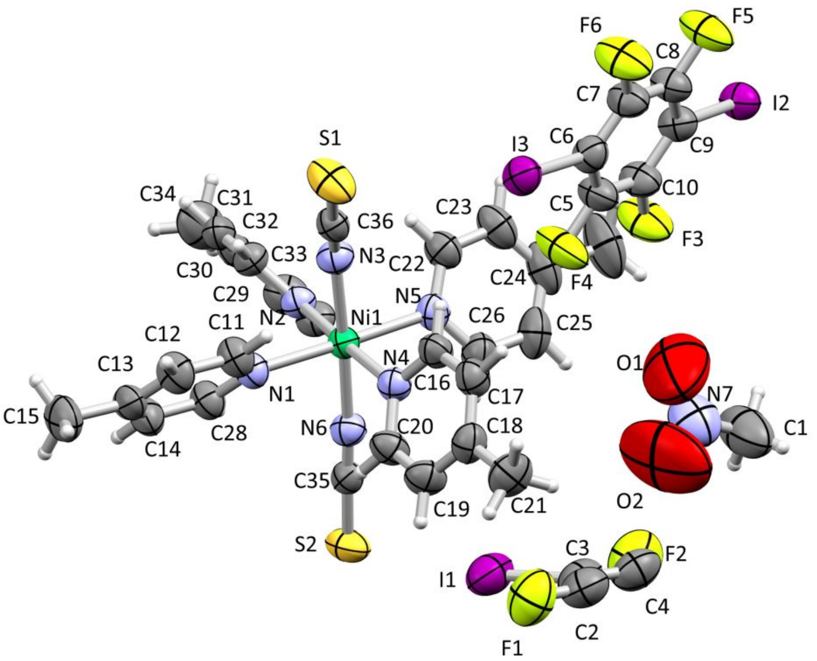

Figure S13. Molecular structure of (**1**)$_2$(**14tfib**)$_3$(**NMT**)$_2$ showing the atom-labeling scheme. Displacement ellipsoids are drawn at the 50 % probability level and H atoms are shown as small spheres of arbitrary radius.

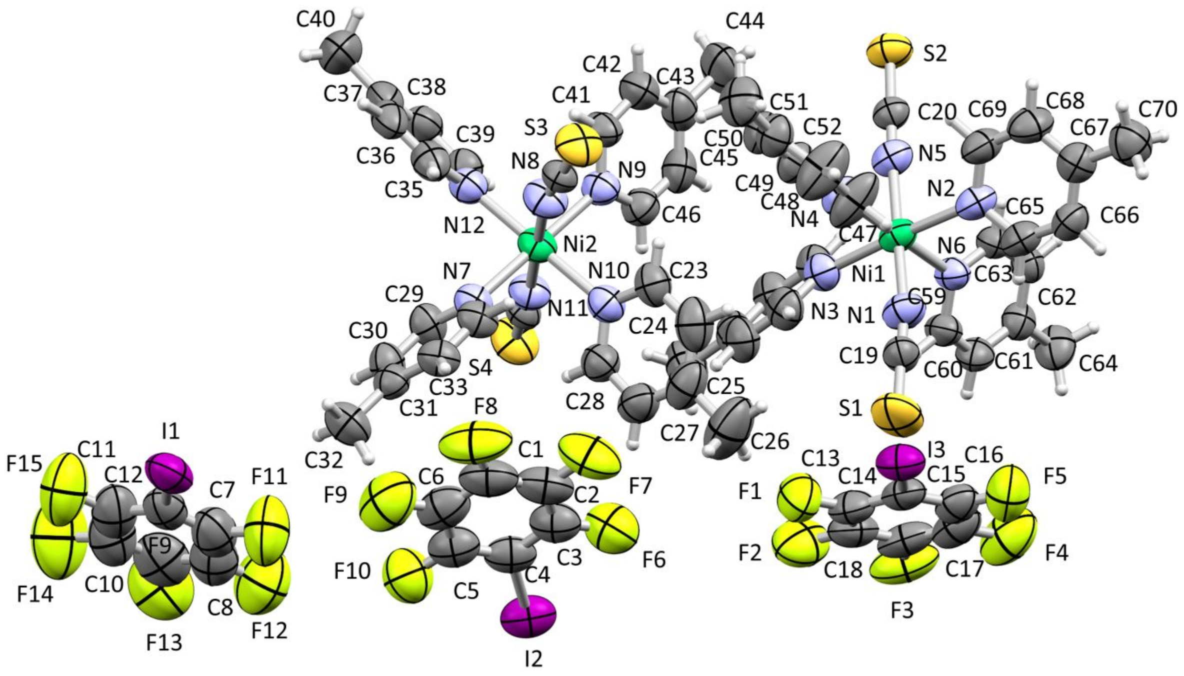

Figure S14. Molecular structure of (**1**)$_2$(**ipfb**)$_3$ showing the atom-labeling scheme. Displacement ellipsoids are drawn at the 50 % probability level and H atoms are shown as small spheres of arbitrary radius.

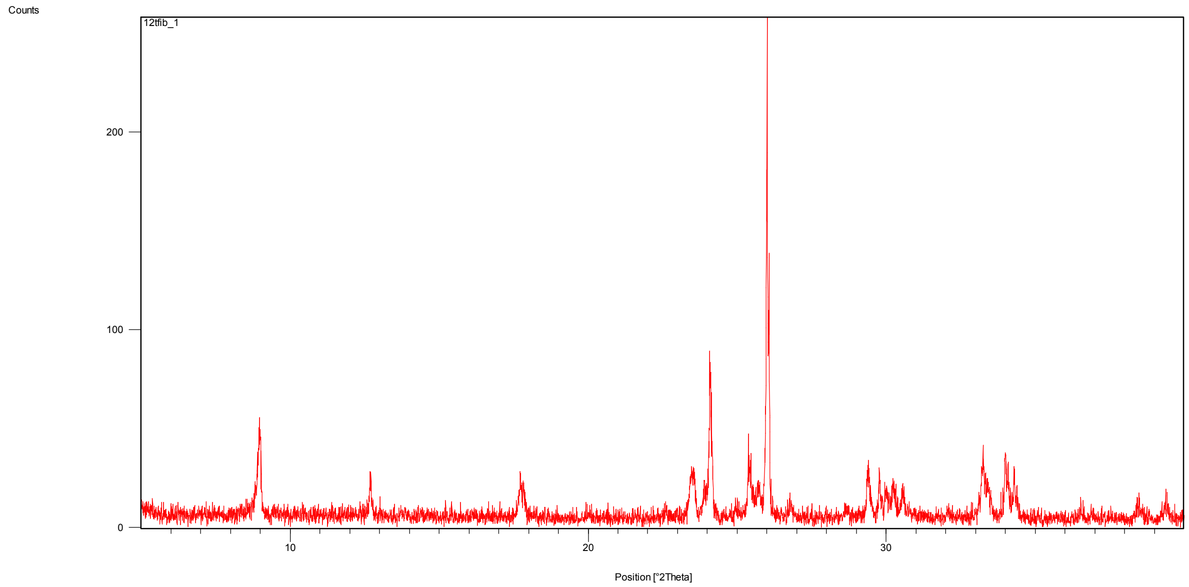

Figure S15. PXRD pattern of **12tfib**.

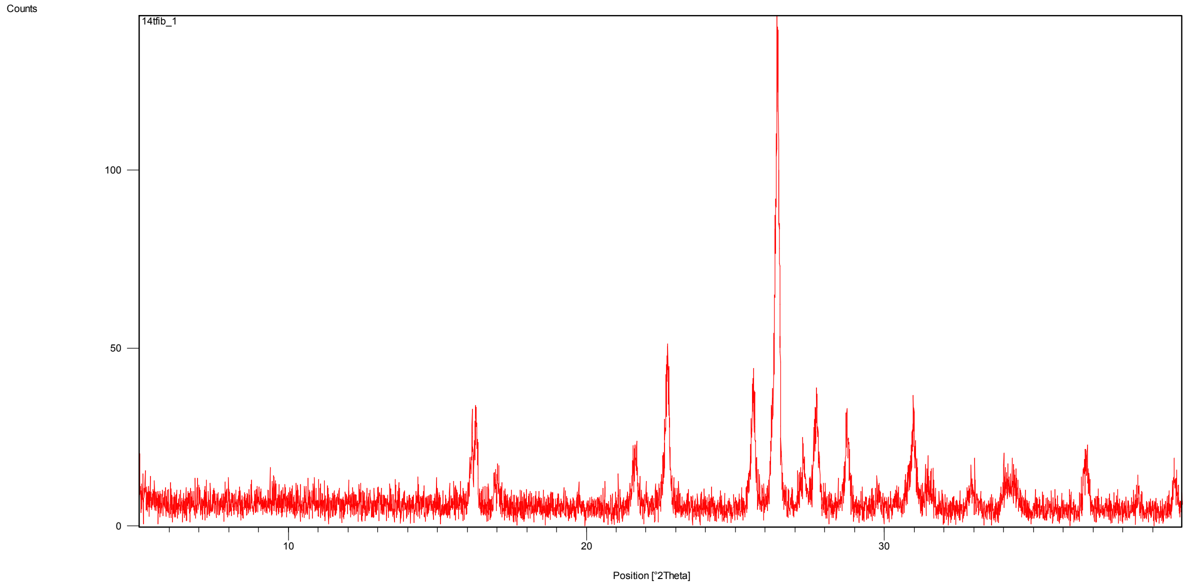

Figure S16. PXRD pattern of **14tfib**.

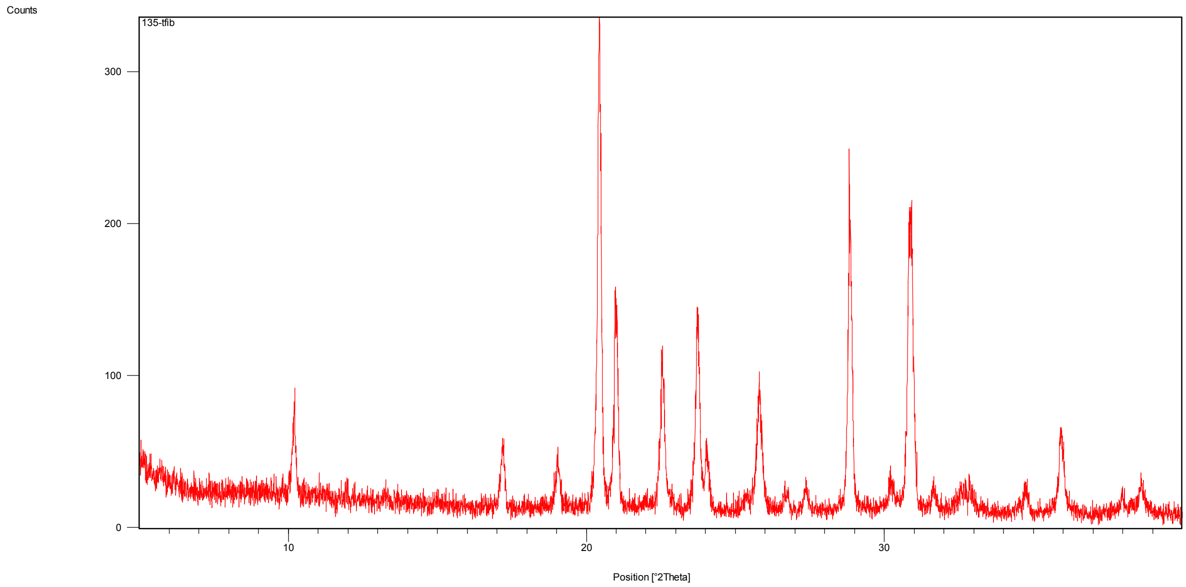

Figure S17. PXRD pattern of **135tfib**.

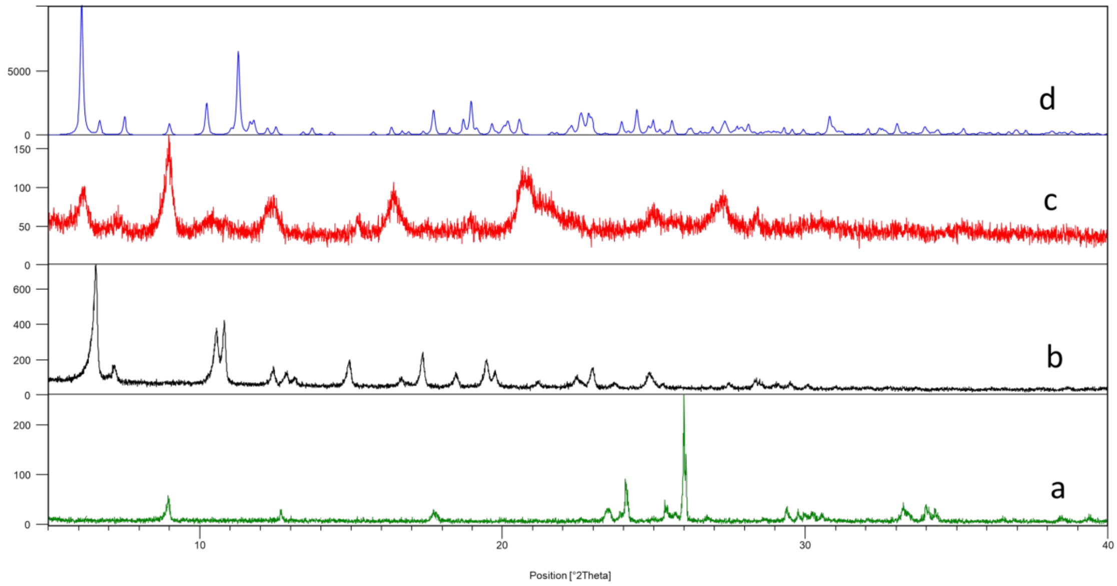

Figure S18. PXRD patterns of: a) **12tfib**, b) **1**, c) product obtained by grinding **12tfib** and **1** in a 1:1 stoichiometric ratio d) calculated pattern from (**1**)(**12tfib**) single crystal data.

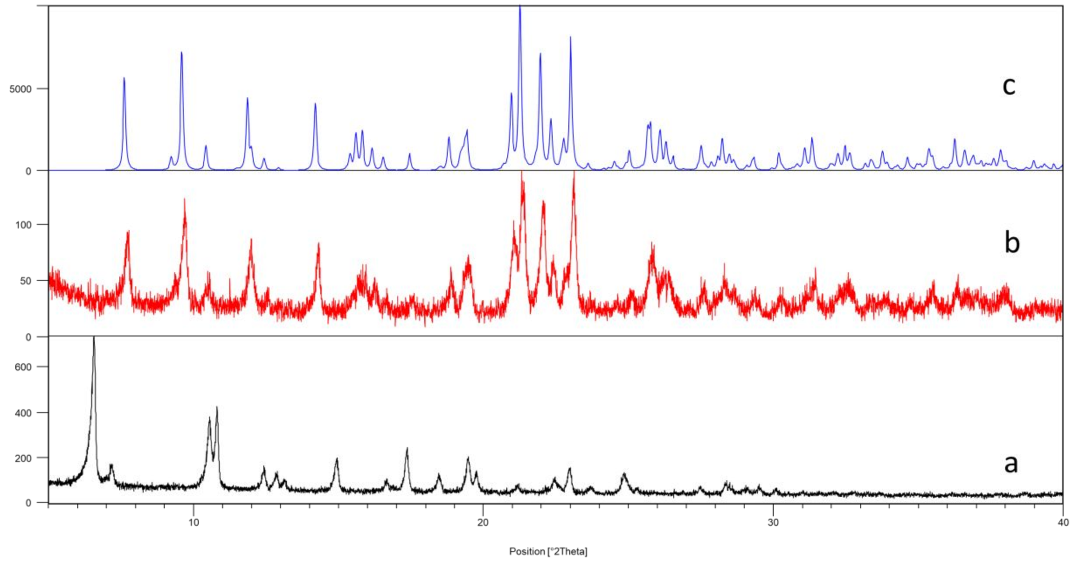

Figure S19. PXRD patterns of: a) **1**, b) product obtained by grinding **13tfib** and **1** in a 1:2 stoichiometric ratio, c) calculated pattern from (**1**)(**13tfib**)$_2$ single crystal data.

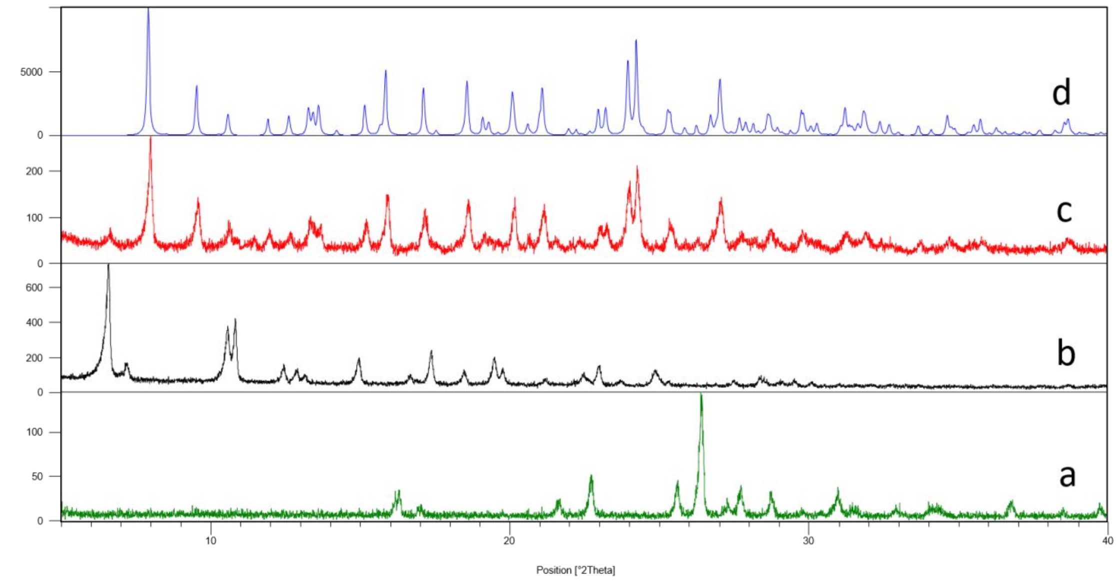

Figure S20. PXRD patterns of: a) **14tfib**, b) **1**, c) product obtained by grinding **14tfib** and **1** in a 1:2 stoichiometric ratio, d) calculated pattern from (**1**)(**14tfib**)$_2$ single crystal data.

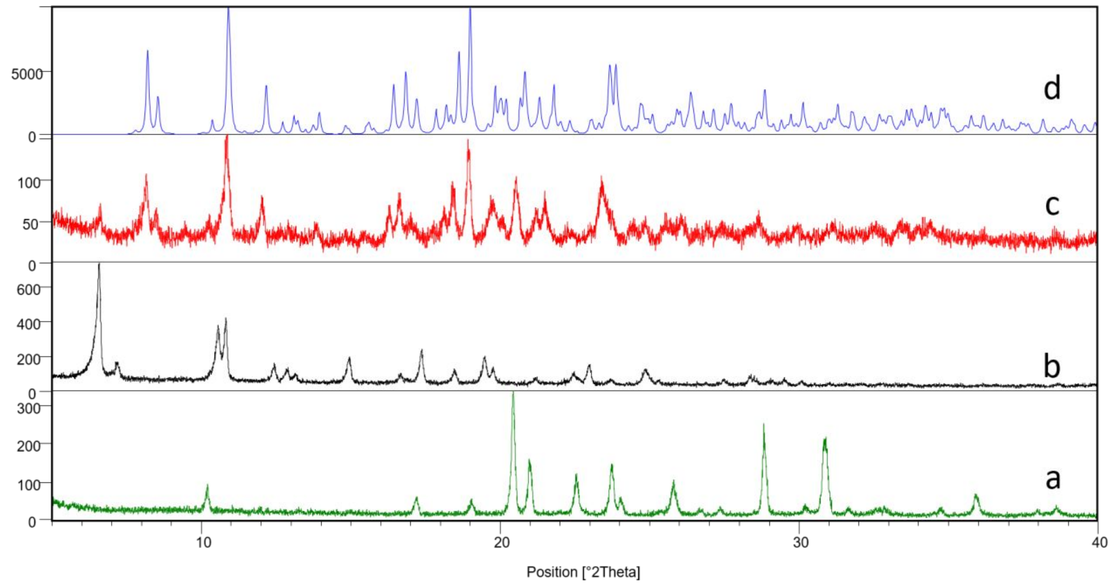

Figure S21. PXRD patterns of: a) **135tfib**, b) **1**, c) product obtained by grinding **135tfib** and **1** in a 1:2 stoichiometric ratio, d) calculated pattern from (**1**)(**135tfib**)$_2$ single crystal data.

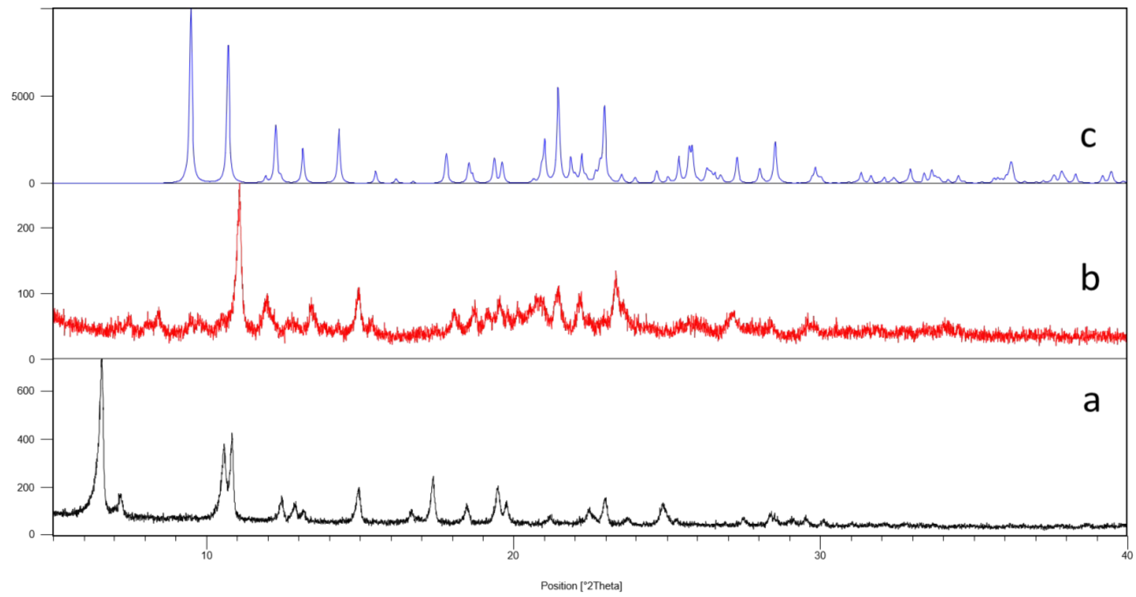

Figure S22. PXRD patterns of: a) **1**, b) product obtained by grinding **ipfb** and **1** in a 1:2 stoichiometric ratio, c) calculated pattern from (**1**)$_2$(**ipfb**)$_3$ single crystal data.

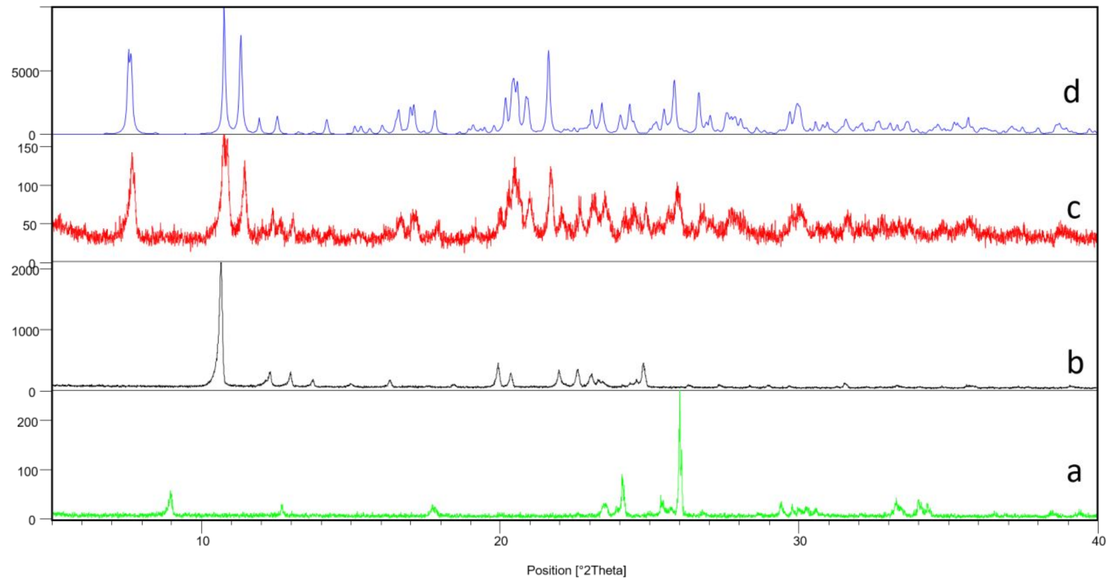

Figure S23. PXRD patterns of: a) **12tfib**, b) **2**, c) product obtained by grinding **12tfib** and **2** in a 1:1 stoichiometric ratio, d) calculated pattern from (**1**)(**12tfib**)$_2$ single crystal data.

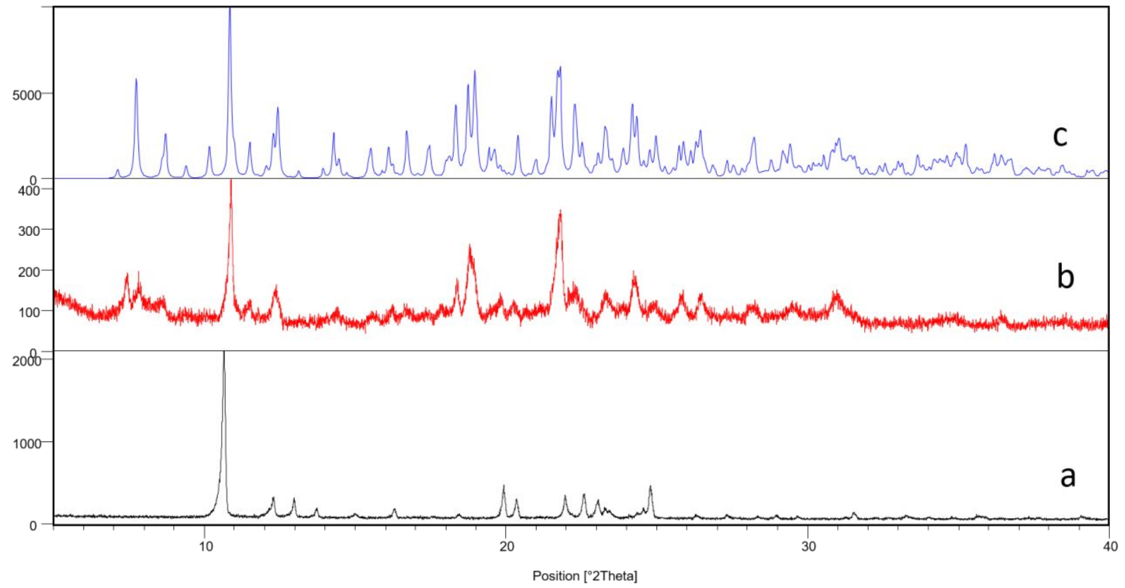

Figure S24. PXRD patterns of: a) **2**, b) product obtained by grinding **13tfib** and **2** in a 2:3 stoichiometric ratio, c) calculated pattern from (**2**)$_2$(**13tfib**)$_3$ single crystal data.

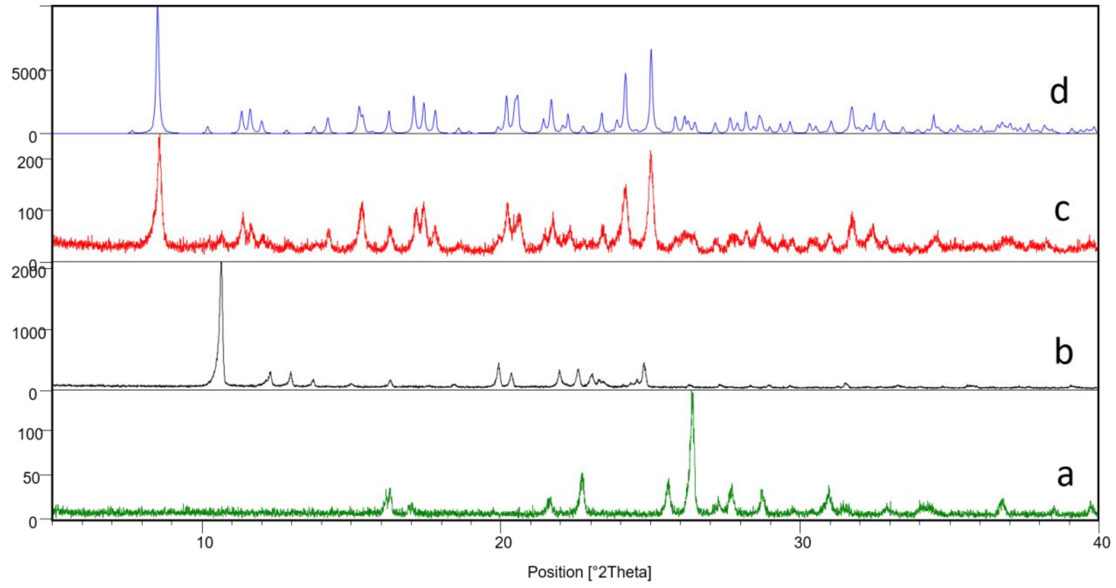

Figure S25. PXRD patterns of: a) **14tfib**, b) **2**, c) product obtained by grinding **14tfib** and **2** in a 1:2 stoichiometric ratio, d) calculated pattern from (**2**)$_2$(**14tfib**)$_3$ single crystal data.

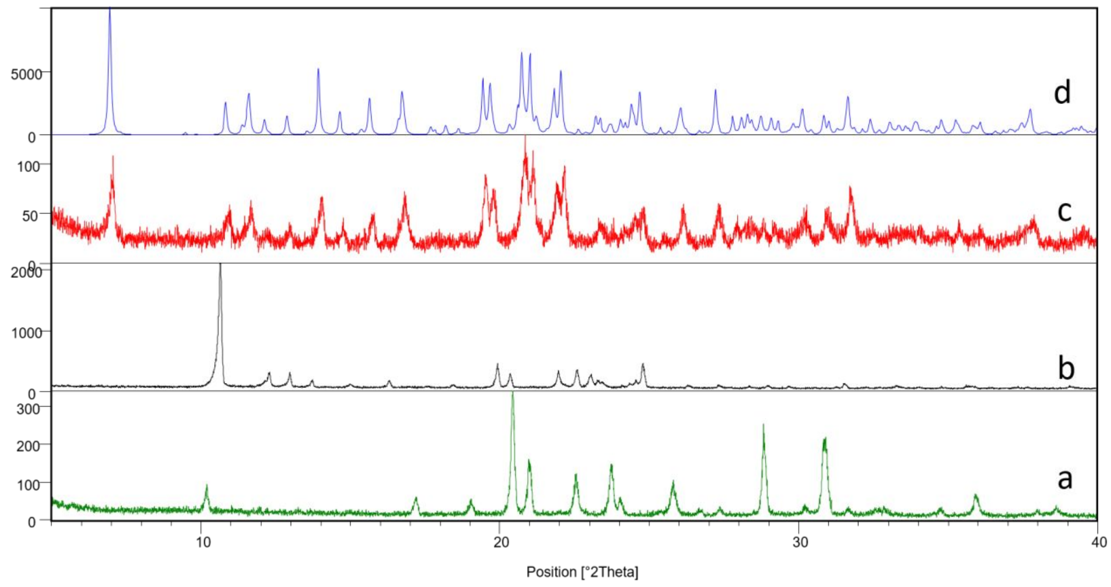

Figure S26. PXRD patterns of: a) **135tfib**, b) **2**, c) product obtained by grinding **135tfib** and **2** in a 1:1 stoichiometric ratio, d) calculated pattern from (**2**)(**135tfib**) single crystal data.

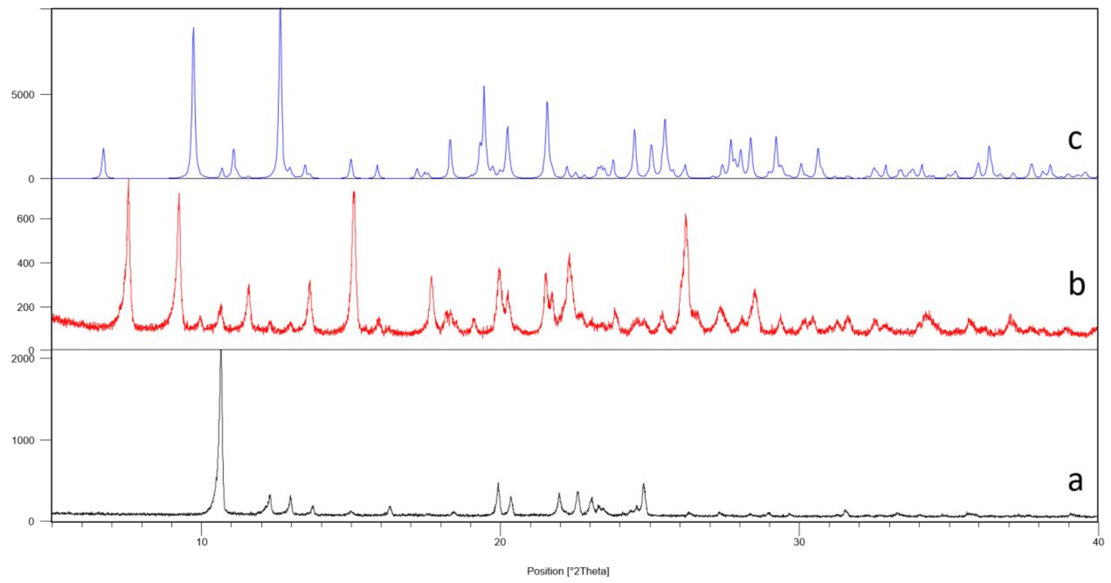

Figure S27. PXRD patterns of: a) **2**, b) product obtained by grinding **ipfb** and **2** in a 1:2 stoichiometric ratio, c) calculated pattern from (**1**)(**ipfb**)$_2$ single crystal data.

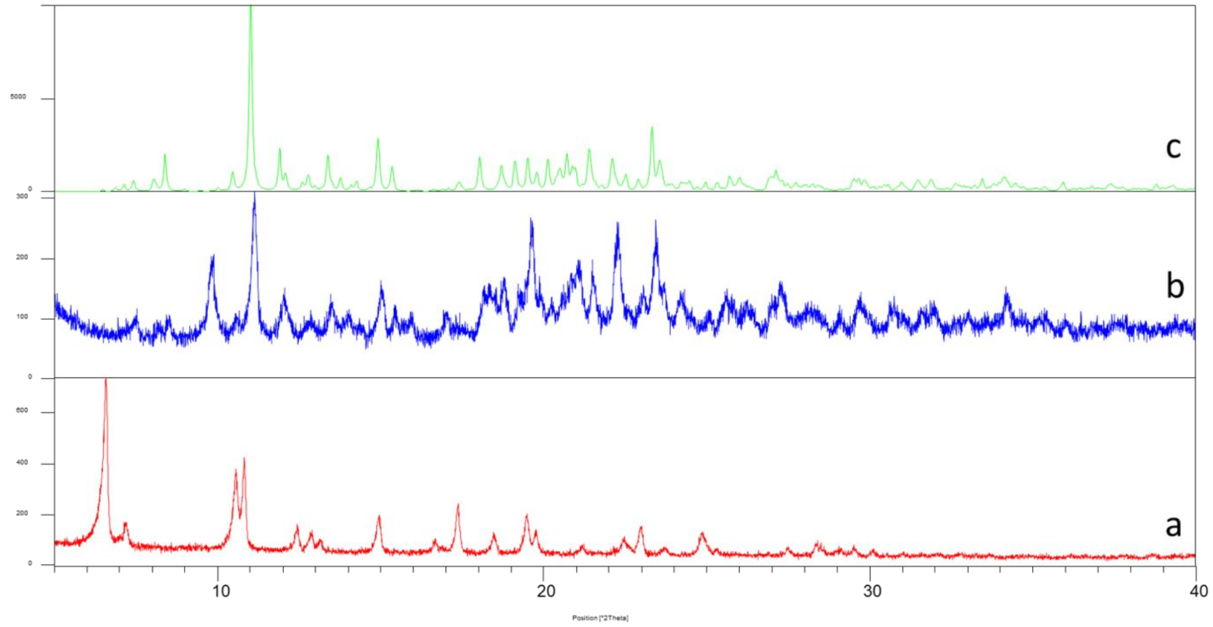

Figure S28. PXRD patterns of: a) **1**, b) product obtained by vapor sorption experiments of **ipfb** and **1**, c) calculated pattern from (**1**)$_2$(**ipfb**)$_3$ single crystal data

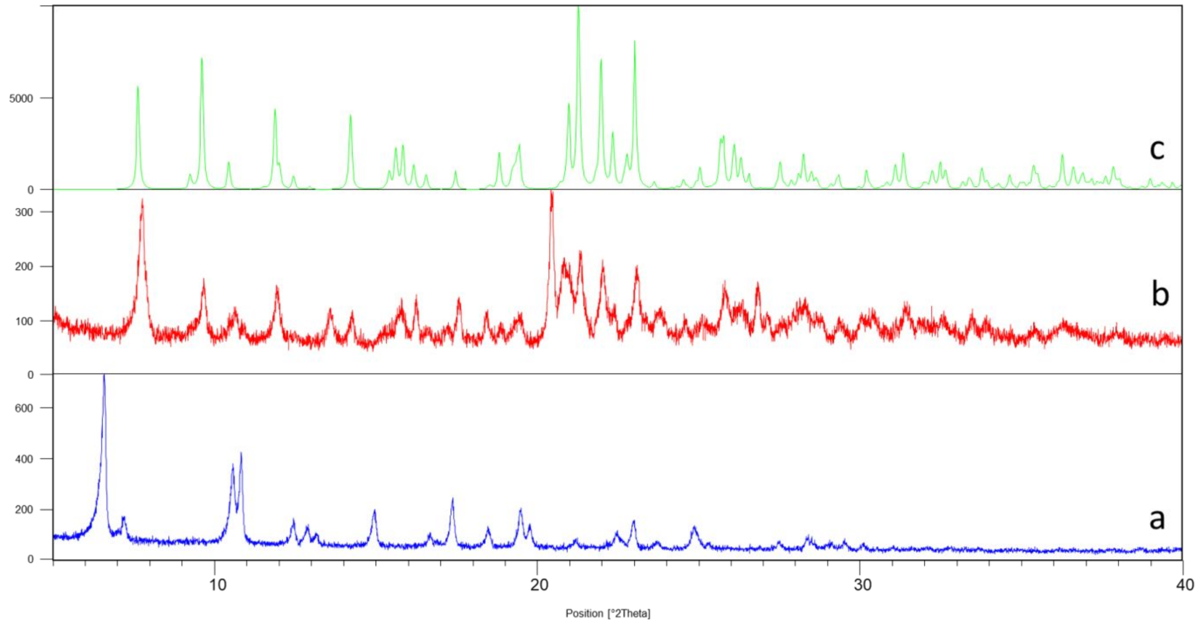

Figure S29. PXRD patterns of: a) **1**, b) product obtained by vapor sorption experiments of **13tfib** and **1**, c) calculated pattern from (**1**)(**13tfib**)$_2$ single crystal data.

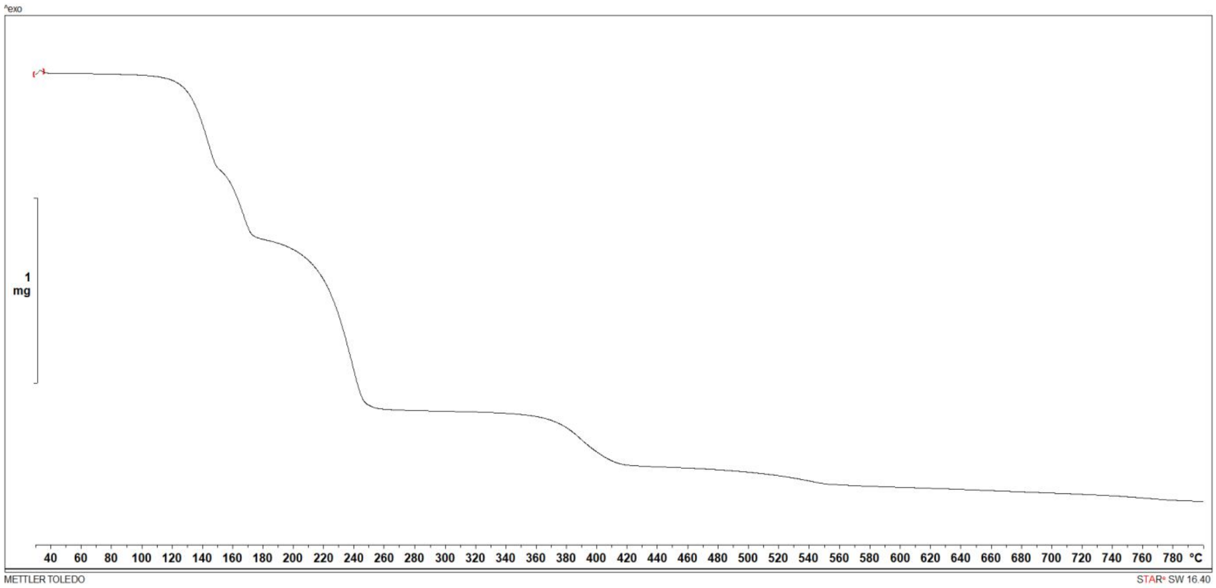

Figure S30. TG curve of **1**.

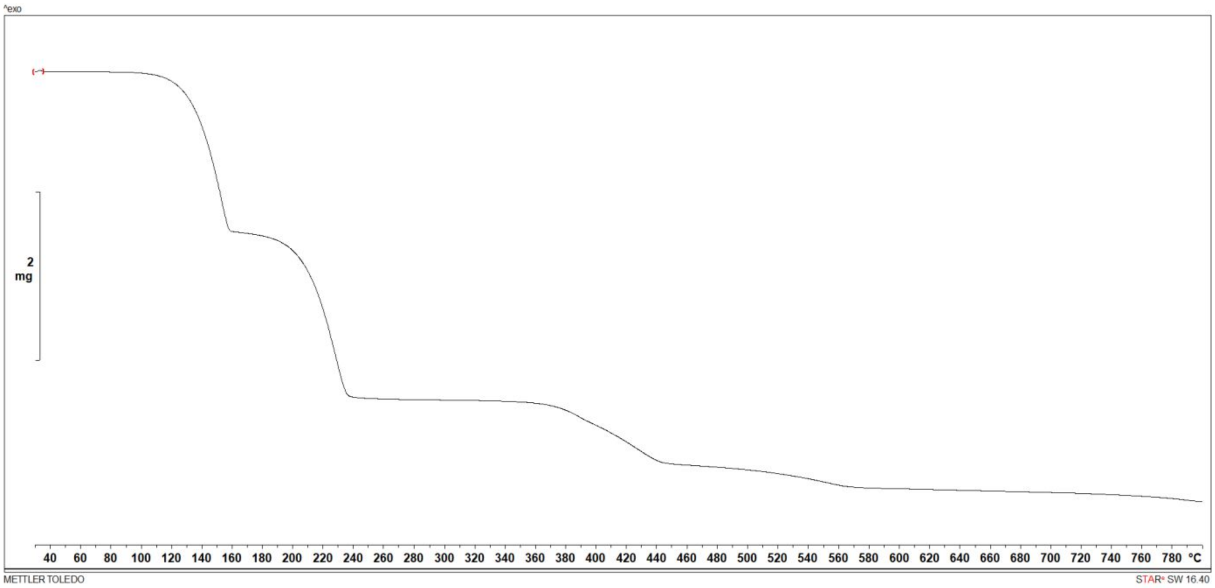

Figure S31. TG curve of **2**.

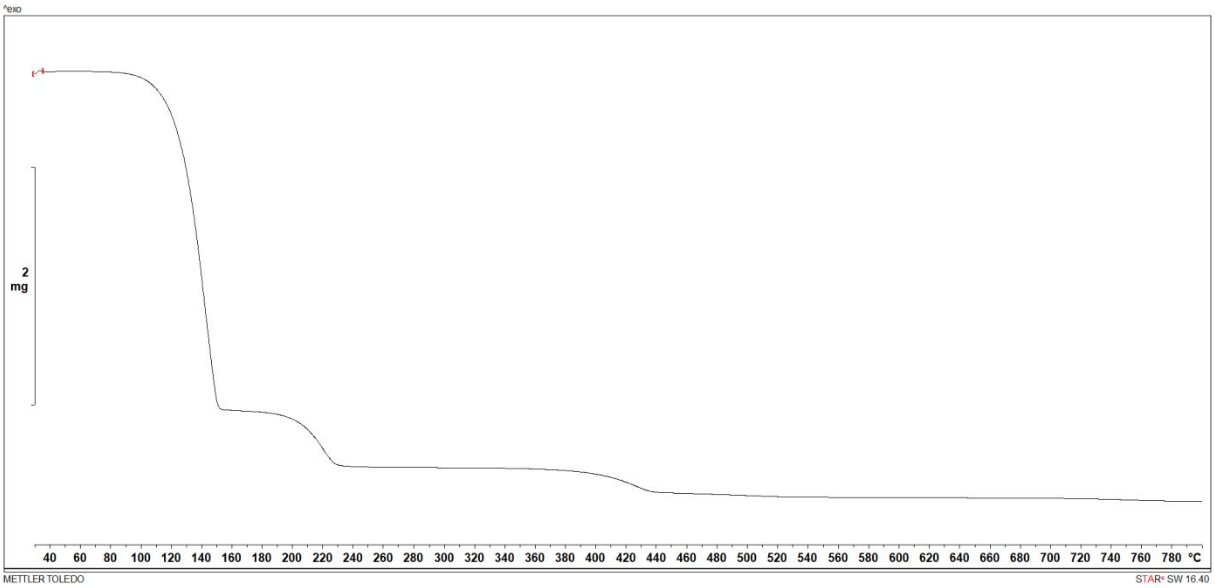

Figure S32. TG curve of the cocrystals (**1**)(**12tfib)**.

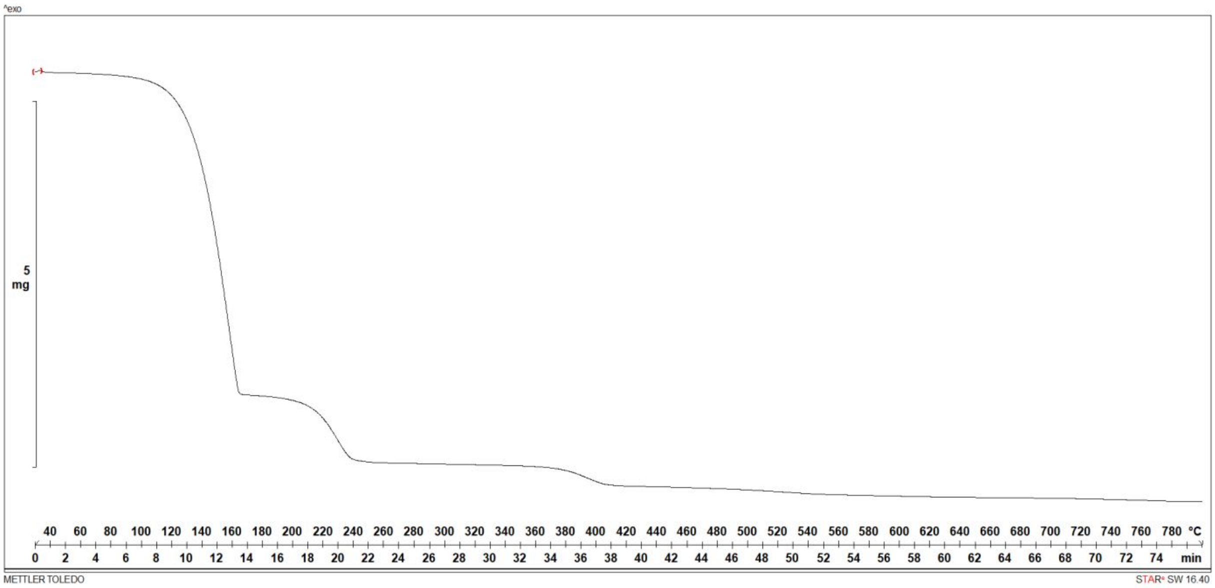

Figure S33. TG curve of the cocrystals (**1**)(**13tfib)**$_2$.

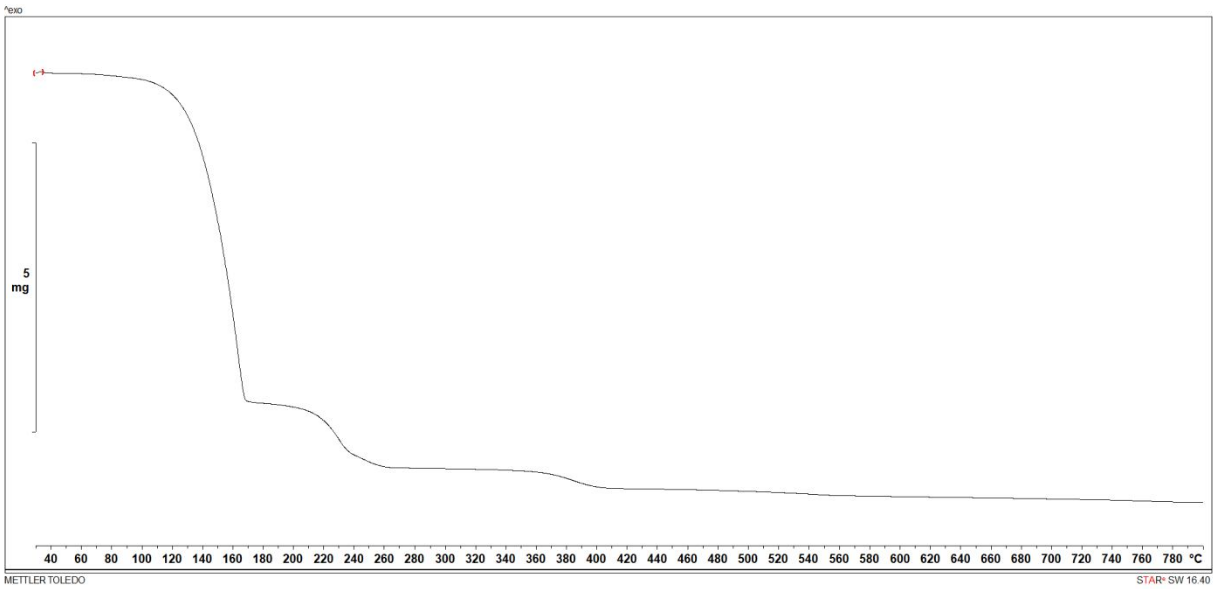

Figure S34. TG curve of the cocrystals (**1**)(**14tfib)**$_2$.

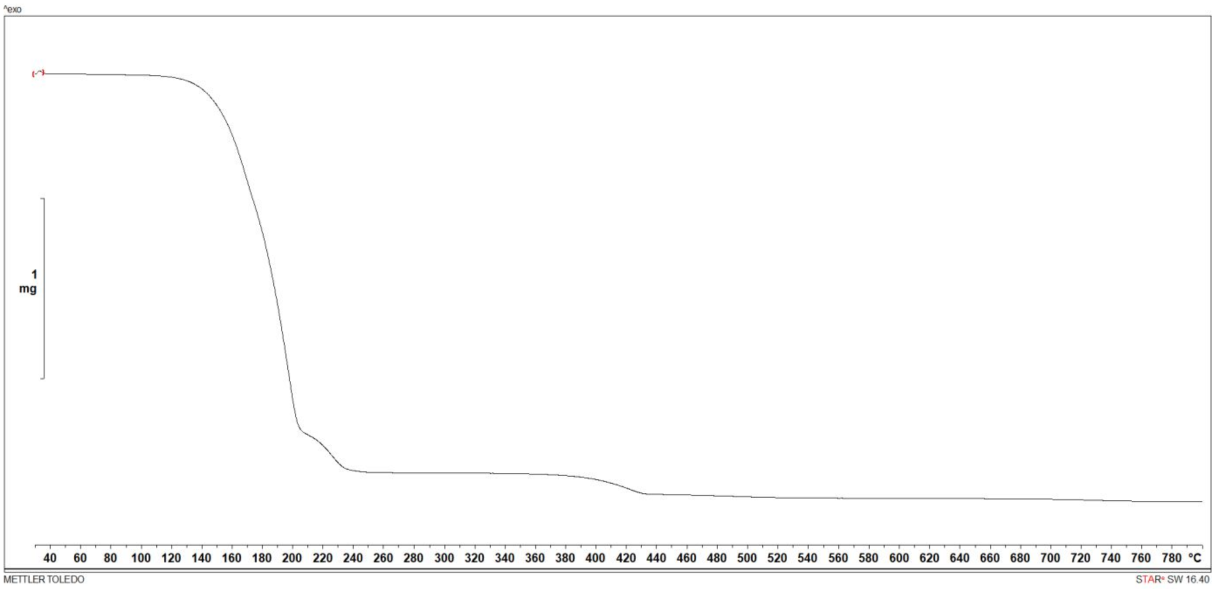

Figure S35. TG curve of the cocrystals (**1**)(**135tfib)**$_2$.

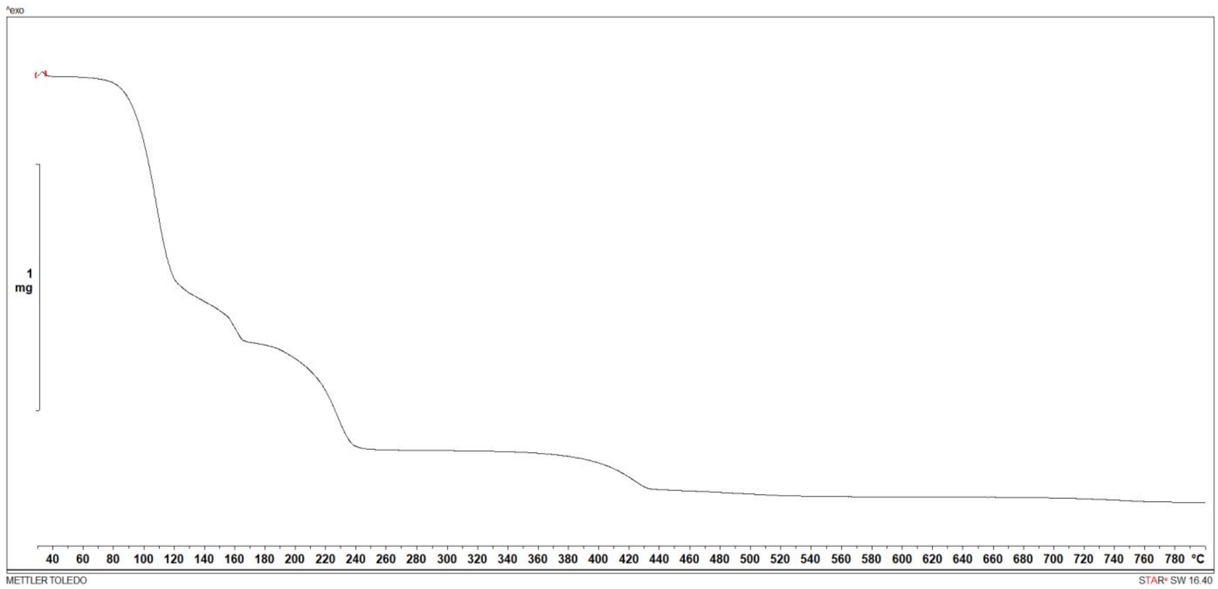

Figure S36. TG curve of the cocrystals (**1**)(**ipfb**)$_2$.

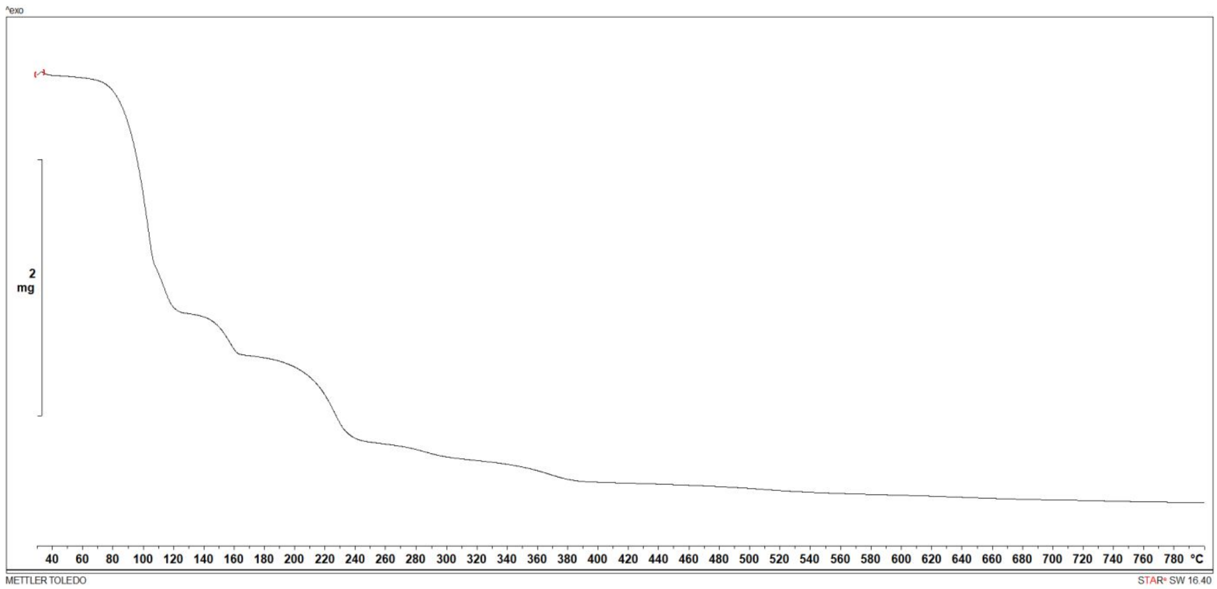

Figure S37. TG curve of the cocrystals (**1**)$_2$(**ipfb**)$_3$.

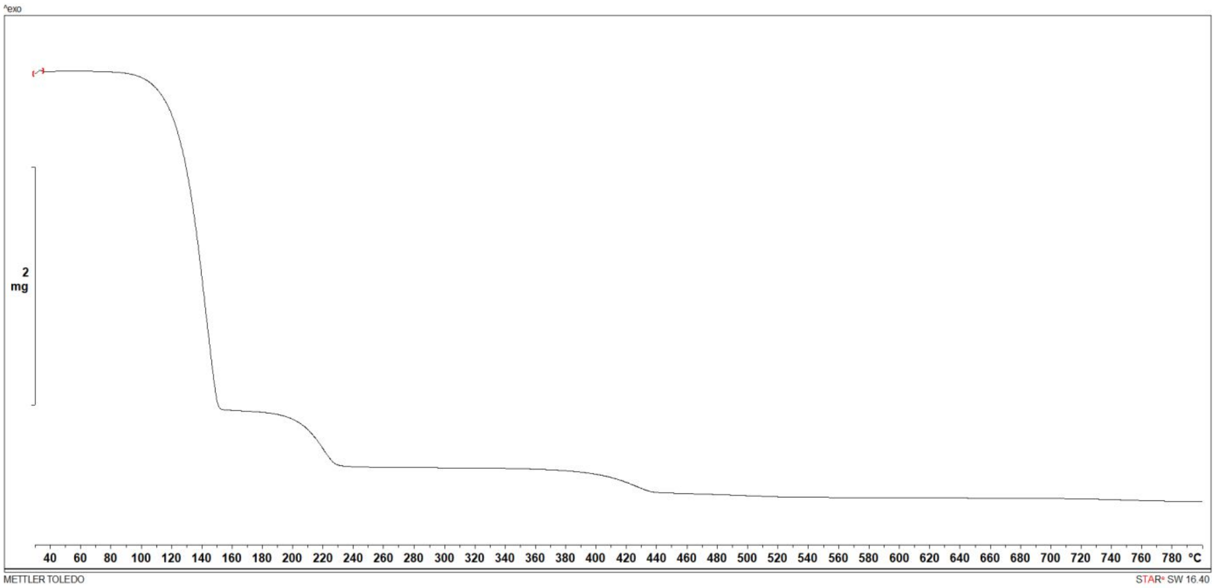

Figure S38. TG curve of the cocrystals (**2**)(**12tfib)$_2$**.

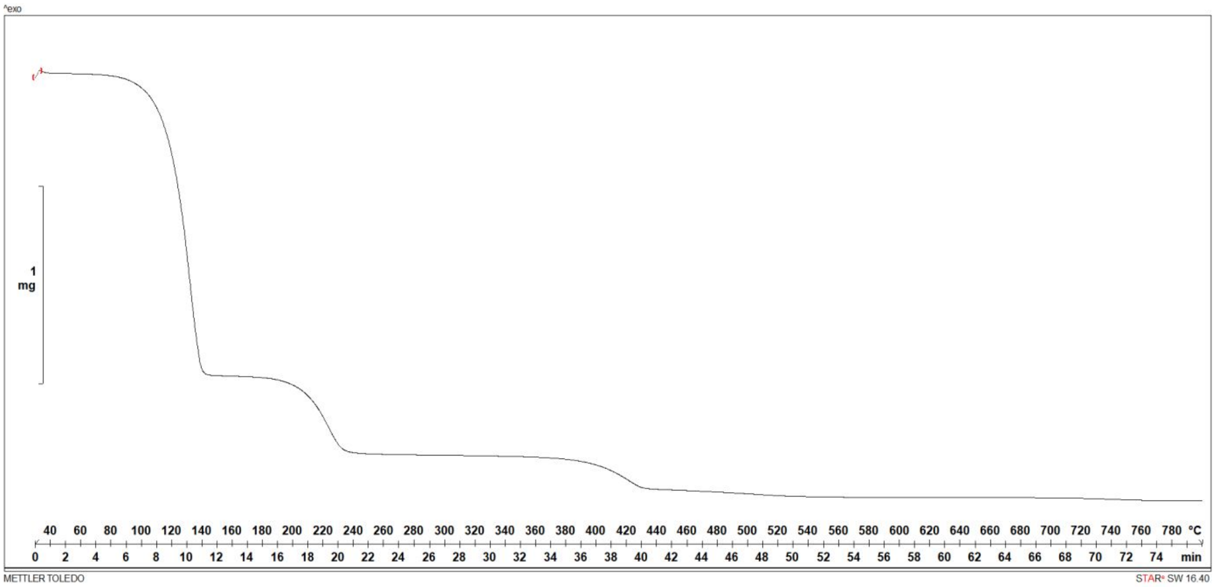

Figure S39. TG curve of the cocrystals (**2**)$_2$(**13tfib)**$_3$.

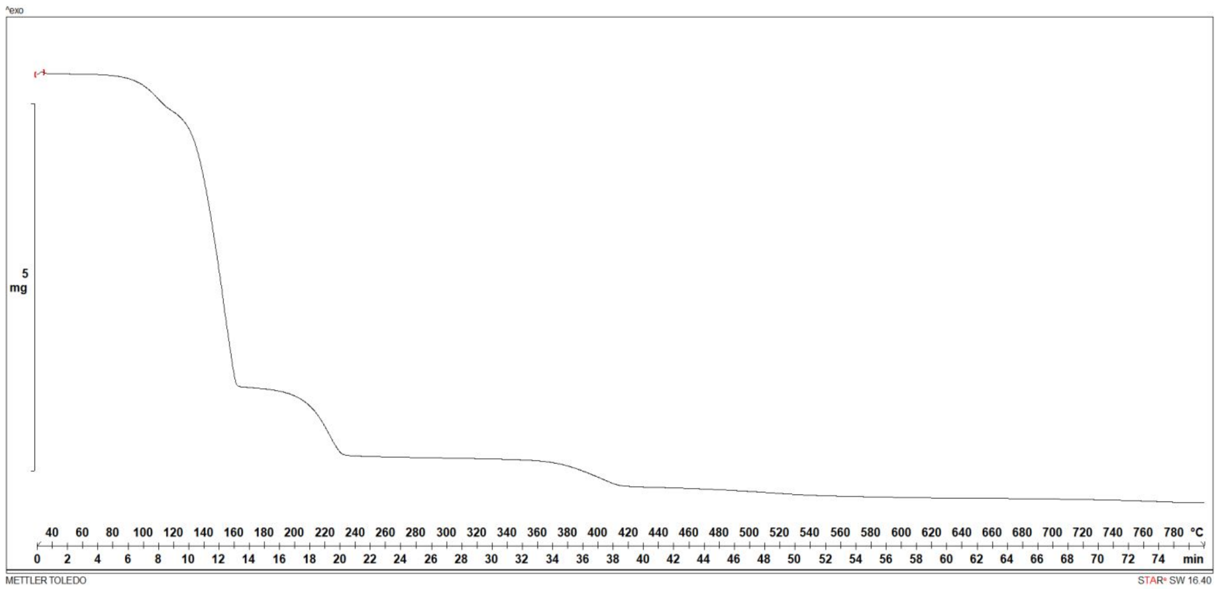

Figure S40. TG curve of the cocrystals (**2**)$_2$(**14tfib**)$_3$.

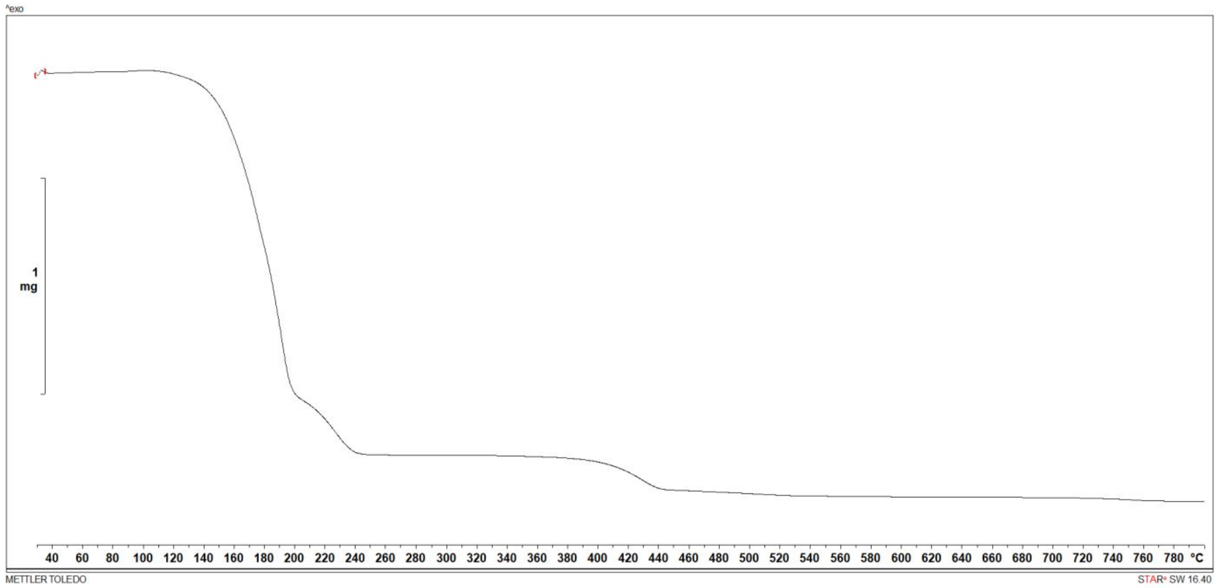

Figure S41. TG curve of the cocrystals (**2**)(**135tfib**).

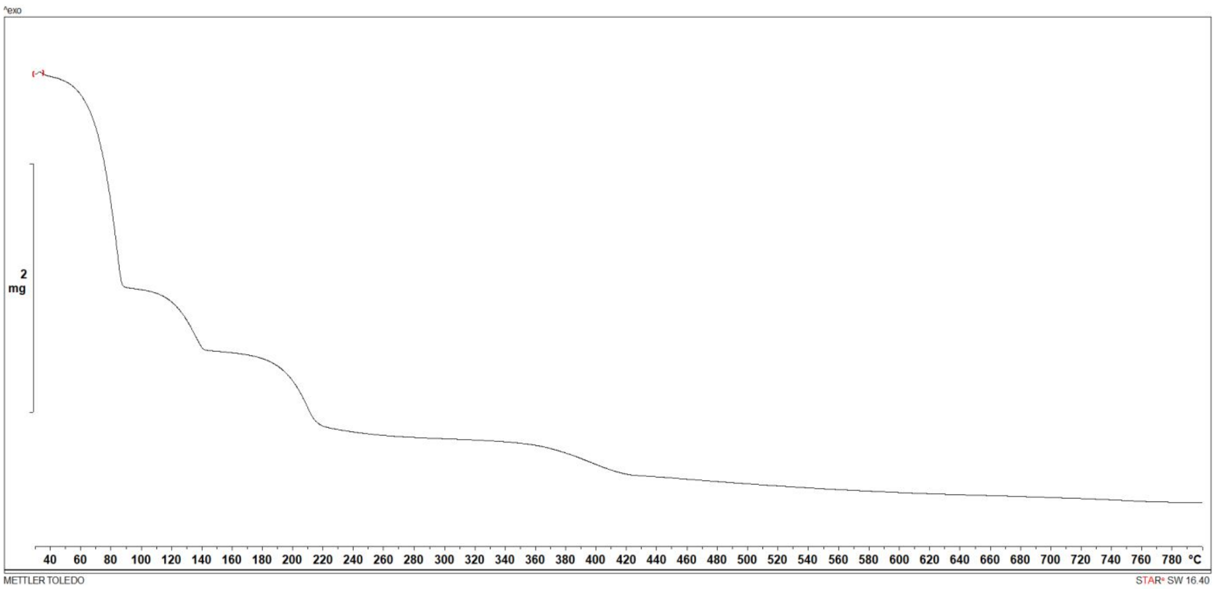

Figure S42. TG curve of the cocrystals (**2**)(**ipfb**)$_2$.

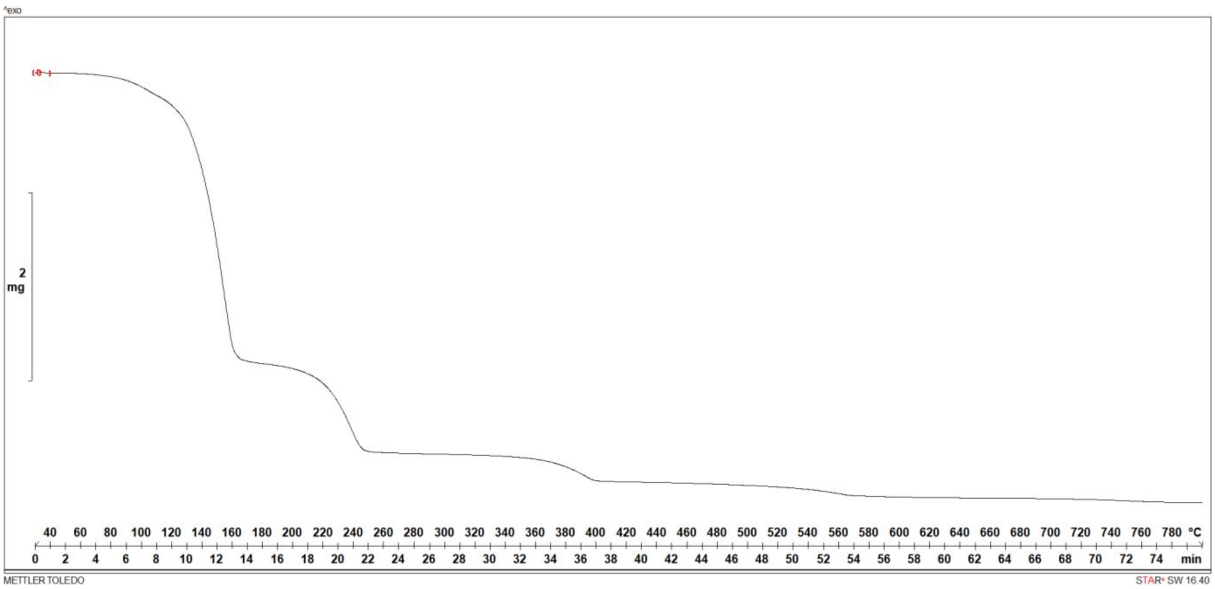

Figure S43. TG curve of the cocrystals (**1**)$_2$(**14tfib**)$_3$(**ACN**)$_2$

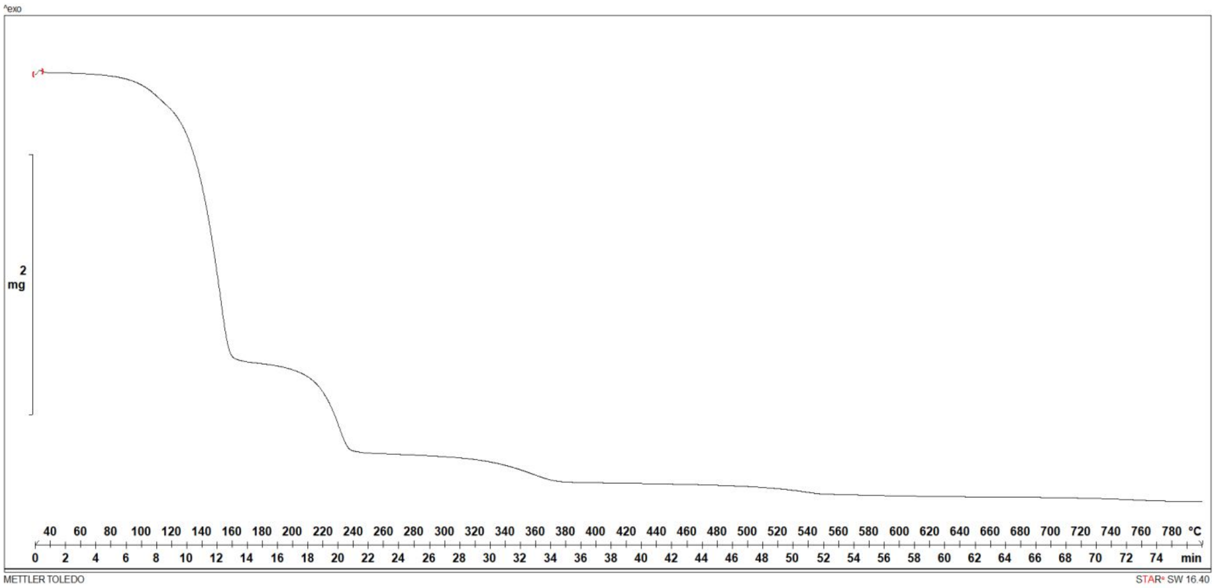

Figure S44. TG curve of the cocrystals (**1**)$_2$(**14tfib**)$_3$(**NMT**)$_2$

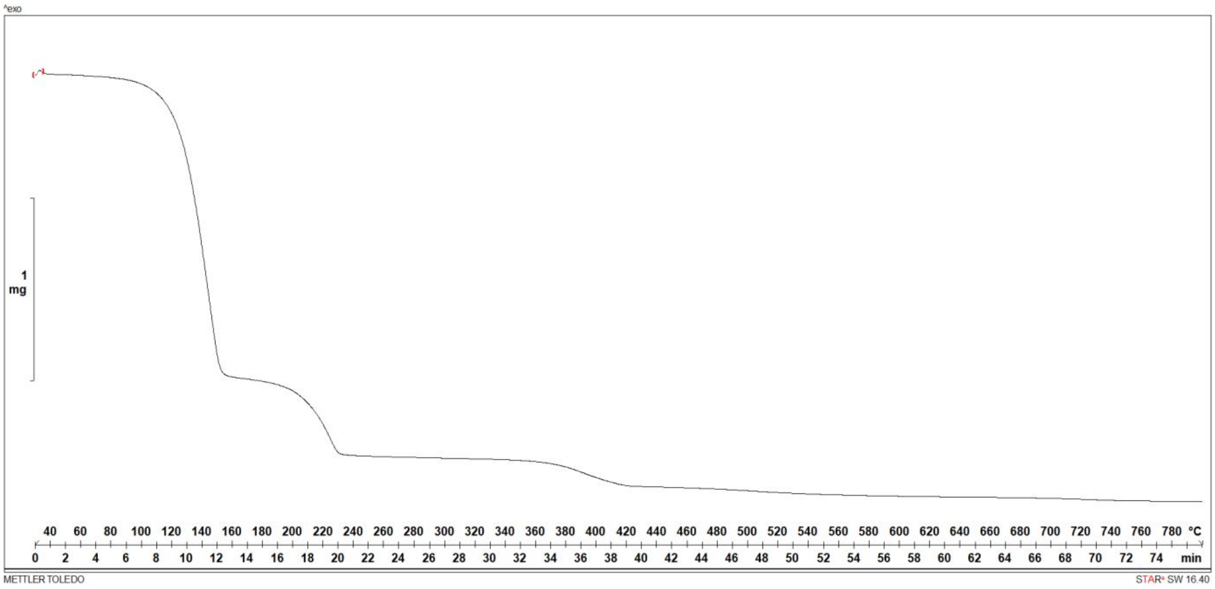

Figure S45. TG curve of the cocrystals (**1**)$_2$(**14tfib**)$_3$(**ACT**)$_2$
